# Choice of adjuvant and antigen composition alters the immunogenic profile of a SARS-CoV-2 subunit vaccine

**DOI:** 10.3389/fddev.2024.1342518

**Published:** 2024-02-07

**Authors:** William R. Lykins, Jeroen Pollet, Jessica A. White, Brian Keegan, Leroy Versteeg, Ulrich Strych, Wen-Hsiang Chen, Raodoh Mohamath, Gabi Ramer-Denisoff, Sierra Reed, Samuel Beaver, Alana Gerhardt, Emily A. Voigt, Mark A. Tomai, Robert Sitrin, Robert K. M. Choy, Frederick J. Cassels, Peter J. Hotez, Maria Elena Bottazzi, Christopher B. Fox

**Affiliations:** ^1^ Access to Advanced Health Institute, Seattle, WA, United States; ^2^ Texas Children’s Hospital Center for Vaccine Development, Houston, TX, United States; ^3^ Department of Pediatrics, National School of Tropical Medicine, Baylor College of Medicine, Houston, TX, United States; ^4^ PATH, Seattle, WA, United States; ^5^ 3M Health Care, St. Paul, MN, United States; ^6^ Department of Biology, Baylor University, Waco, TX, United States; ^7^ James A. Baker III Institute for Public Policy, Rice University, Houston, TX, United States; ^8^ Department of Global Health, University of Washington, Seattle, WA, United States

**Keywords:** receptor binding domain, adjuvant formulation, SARS-CoV-2, vaccine development, toll-like receptor, bivalent vaccine

## Abstract

**Introduction:** Since their introduction, adjuvanted recombinant subunit vaccines against COVID-19 have played a pivotal role in protecting global populations. Optimizing the immune response’s quality, amplitude, and durability to these vaccines depends on the appropriate adjuvant choice and dose in combination with the selected antigen.

**Methods:** Here, we employed a preclinical mouse model to study the adaptive humoral and cellular immune responses to a SARS-CoV-2 receptor binding domain (RBD) antigen formulated with one of four different immune agonists [GLA, 3M-052, CpG-1826 (CpG), and dmLT], in combination with one of two different immune-stimulating formulations, a stabilized squalene emulsion (SE) or aluminum hydroxide (Alum). Using a weighted desirability index, we established an immunogenicity ranking for each adjuvant in combination with the RBD antigen.

**Results:** We found that formulations of the RBD with Alum in combination with either 3M-052 or CpG led to at least a 2-log increase in serum IgG production and a 1.3- to 2.2-log increase in the number of bone marrow-derived antibody-secreting cells compared to the RBD formulated with Alum without an additional agonist. In contrast, the RBD formulated with SE in combination with 3M-052 or CpG did not elicit an IgG response greater than the unadjuvanted control. Additionally, RBD formulated with 3M-052 or CpG on Alum generated a 0.8- or 1.6-log lower splenocyte IL-5 response (a pro-Th2 marker), respectively, than Alum without an additional agonist. When formulated with 3M-052-Alum, a bivalent vaccine containing the original lineage (Wuhan-Hu-1) and the Delta variant (B.1.617.2) RBD antigens led to a more than 2-log increase in neutralizing antibodies against an Omicron variant (B.1.1.529) pseudovirus in vaccinated animals compared to animals that received the monovalent RBD antigen.

**Discussion:** Our results suggest that optimal immune responses to subunit antigens may be achieved through an orthogonal approach that applies adjuvant formulation, antigen combination, and advances in rational vaccine development techniques.

## 1 Introduction

Subunit vaccines have played a pivotal role in the global response to the SARS-CoV-2 pandemic (Heidary et al., 2022). Compared to other approved whole virus or RNA-based vaccines, adjuvanted subunit vaccines have several advantages in terms of manufacturing cost, stability, and public acceptance that make them especially attractive options for use in low- to middle-income countries (Pollet et al., 2021; Hotez et al., 2023). Currently, several emergency-authorized or approved adjuvanted subunit vaccines against SARS-CoV-2 are in use around the world, such as Corbevax, IndoVac, and Nuvaxovid. Corbevax and IndoVac were developed by Biological E. Limited in India and PT Bio Farma in Indonesia, respectively, and in collaboration with Texas Children’s Hospital Center for Vaccine Development, with almost 100 million doses administered to date (Hotez et al., 2023). Both use slightly modified versions of the wild-type (wt) SARS-CoV-2 spike receptor binding domain (RBD) as an antigen, RBD219-N1C1 or RBD203-N1, respectively, formulated with aluminum hydroxide (Alum) and the TLR9 agonist CpG-1018 (Hotez et al., 2023). Corbevax and IndoVac build upon technologies that have been successfully employed in the production of recombinant hepatitis B vaccine antigens and employ an adjuvant that does not require frozen storage, in contrast to the currently available mRNA vaccines based on lipid nanoparticles (Kloczewiak et al., 2022). The success of Corbevax and IndoVac motivates the further development of low-cost vaccine options that can 1) generate protective and durable humoral and cellular responses, 2) protect against a wider range of viral variants, and 3) provide improved protection at the mucosal site of infection.

The RBD203-N1 antigen is a fragment of the wt SARS-CoV-2 spike protein RBD. The RBD attaches to ACE-2 in human cells mediating infection (Tai et al., 2020). Compared to the RBD219-N1C1, RBD203-N1 was found to have increased production yields while maintaining equivalent biophysical and immunological properties (Chen et al., 2022; Pollet et al., 2022). The immunogenicity of this family of RBD antigens has primarily been tested using Alum as an adjuvant and formulation system, with or without the addition of either the TLR9 agonist CpG-1018 or the TLR7/8 agonist 3M-052 (Chen et al., 2020; 2022; Pino et al., 2021; Pollet et al., 2022). It is of interest to understand how RBD203-N1 (hereafter RBD) interacts with other common adjuvant formulations and agonists, such as squalene emulsions, the synthetic TLR4 agonist glucopyranosyl lipid adjuvant (GLA), the murine TLR9 agonist CpG-1826 (CpG), the TLR7/8 agonist 3M-052, and the detoxified *Escherichia coli* double mutant heat-labile toxin (dmLT) LT(R192G/L211A), all of which are in either clinical or commercial stage use, to determine if there are synergies, or how these formulations influence the immune phenotype of the RBD vaccine.

To this end, we tested the RBD203-N1 antigen in combination with a range of adjuvant formulations to identify lead adjuvant candidates, and then tested those in combination with a bivalent antigen (Wuhan and Delta B.1.617.2) against three different SARS-CoV-2 variants (Wuhan strain, Delta B.1.617.2, and Omicron B.1.1.529). We present a methodical down selection of optimal adjuvant compositions for use with the RBD subunit antigen and show how this adjuvant formulation interacts with bivalency and the physical characteristics of the antigen.

## 2 Materials and methods

### 2.1 Raw materials

Recombinant RBD203-N1 wt (Wuhan-Hu-1) and Delta (B.1.617.2) variants were provided by Texas Children’s Hospital Center for Vaccine Development (Houston, TX). dmLT (Vaccine Ontology Identifier VO_0005329) was provided by PATH (Seattle, WA). Aluminum hydroxide (Alhydrogel 2%) was procured from Croda (Princeton, NJ; Cat# 21645-51-2). Shark squalene (Cat# S3626) was procured from Sigma-Aldrich (St. Louis, MO). Alpha-tocopherol (vitamin E; Cat# VI135), Poloxamer 188 (Cat# P1169), and glycerin (Cat# G1015) were procured from Spectrum Chemical (New Brunswick, NJ). Egg phosphatidylcholine (egg PC; Cat# 510500), 1,2-dimyristoyl-sn-glycero-3-phosphocholine (DMPC; Cat# 556200), 1,2-dipalmitoyl-sn-glycero-3-phosphocholine (DPPC; Cat# 556300), and 1,2-distearoyl-sn-glycero-3-phospho-rac-glycerol (DSPG; Cat# 840465X) were procured from Lipoid (Ludwigshafen, Germany). GLA-AF (Vaccine Ontology Identifier VO_0005424), 3M-052-AF (Vaccine Ontology Identifier VO_0005474), and 0.9% (w/v) saline were acquired in-house. CpG-1826 (CpG; Cat# tlrl-1826-1) was procured from InvivoGen (San Diego, CA). Unless otherwise noted, all aqueous buffers were produced using Milli-Q water (MilliporeSigma, Burlington, MA). All other materials (unless otherwise noted) were acquired from Fisher Scientific (Hampton, NH).

### 2.2 Adjuvant production and vaccine mixing

All adjuvants and vaccines were prepared under aseptic conditions as previously described. Briefly, Alum-containing adjuvants were prepared by carefully resuspending Alhydrogel (2%) via gentle hand swirling. Alum and an agonist (3M-052-AF, GLA-AF, CpG, or dmLT) were then combined and diluted to the appropriate concentration using sterile 0.9% (w/v) saline. Alum-agonist mixtures were allowed to adsorb for at least 10 min at room temperature (RT) before the addition of the antigen (Fox et al., 2016).

Stabilized squalene emulsion (SE)-containing adjuvants were prepared via microfluidization as previously described (Orr et al., 2013; Misquith et al., 2014). Briefly, a 25 mM ammonium phosphate buffer with Poloxamer 188 as a surfactant and glycerol as a tonicity agent was combined with the homogenous oil phase (squalene and DMPC or egg PC in the case of 3M-052-SE), using a high-shear mixer (Silverson Machines, East Longmeadow, MA) to form a crude emulsion. The emulsion was further downsized using a Microfluidics (Newton, MA) M110P Microfluidizer at 30,000 psi to a final approximate particle size of 100 ± 5 nm d. The final composition of SE before mixing was 4% (w/v) squalene, 0.76% (w/v) DMPC or egg PC, 0.036% (w/v) Poloxamer 188, and 2.3% (w/v) glycerin, in a 25 mM ammonium phosphate buffer (pH 5.1). GLA-SE (Vaccine Ontology Identifier VO_0005420) and 3M-052-SE (Vaccine Ontology Identifier VO_0005386) were produced similarly, incorporating GLA or 3M-052 into the oil phase at a final concentration of 100 or 40 μg/mL, respectively, prior to microfluidization. 3M-052-SE also contained 0.02% (w/v) alpha-tocopherol as an antioxidant, incorporated into the oil phase. CpG and dmLT were incorporated immediately before antigen mixing.

RBD aliquots were individually frozen and stored at −80°C until use. RBD aliquots were thawed at RT and diluted to 140 μg/mL in 0.9% (w/v) saline. Vaccine samples were mixed at a 1:1 (v:v) adjuvant:RBD ratio and stored on ice until use. All vaccine samples were mixed and used within 1 h of thawing the RBD.

### 2.3 Animal use and procedures

BALB/c mice were purchased from The Jackson Laboratory (Harbor, ME). Experimental groups consisted of equal numbers of 6–8-week-old male and female mice. All presented animal experiments were divided in half and vaccinations/harvests staggered 1 week apart to reduce operator burden. Mice were immunized by intramuscular injection of 100 μL total volume (50 μL in each hind leg) of vaccine as indicated on Day 0 and Day 21. Serum and bronchoalveolar lavage (BAL)-based assays for each study (e.g., antibody titer, pseudovirus neutralization) were performed for all animals simultaneously using frozen serum and BAL samples, respectively. Assays relying on live cells (e.g., enzyme-linked immunosorbent spot [ELISpot]) were performed at the time of tissue harvest. All animal experiments were performed in accordance with national and institutional guidelines for animal care of laboratory animals and were approved by the Bloodworks Northwest Research Institute’s Institutional Animal Care and Use Committee (Seattle, WA).

### 2.4 Serum and tissue collection

Peripheral blood was collected via the retro-orbital route under light isoflurane sedation on Day 0 and Day 21 and via cardiac puncture on Day 42. Serum was stored at −80°C until analysis. Mice were euthanized on Day 42 through carbon dioxide inhalation, followed by cervical dislocation. Spleen and bone marrow tissues were harvested, and BAL was performed. Spleens were manually disrupted, and lymphocytes were isolated via Red Blood Cell (RBC) Lysis Buffer (eBioscience, San Diego, CA, #50-112-9751) and washing. Bone marrow was collected from mouse femurs via centrifugation. Bone marrow-resident lymphocytes were isolated via treatment with RBC lysis buffer and washing. BAL samples were collected through a small incision made in the trachea. A 1.5-mL transfer pipet containing saline was inserted in the incision and used to irrigate the lung cavity. The aspirated fluid was centrifuged to remove bulk mucus and stored at −80°C until analysis.

### 2.5 Serum and BAL antibody ELISA

SARS-CoV-2 RBD203-N1 (RBD)-specific antibodies in serum and BAL samples were quantified by enzyme-linked immunosorbent assay (ELISA). All ELISA and ELISpot wash steps were performed with PBS with 0.1% (w/v) Tween-20 (PBST) unless otherwise stated. Briefly, 384-well plates were coated with 25 μL of 2 μg/mL SARS-CoV-2 RBD in phosphate-buffered saline (PBS) and incubated overnight at 4°C. Plates were then blocked with 70 μL of PBST and 1% (w/v) bovine serum albumin for 2 h at RT. In a separate plate, serum was diluted initially 200–650-fold followed by eleven 3-fold serial dilutions. RBD-coated plates were washed and filled with 20 μL of PBST/well. 5 μL of each serum dilution was transferred to the PBST-containing wells of the RBD-coated plate and then incubated at room temperature with serum for 1 h. Serum-treated plates were washed then probed with 25 μL of goat anti-mouse IgG, IgG1, or IgG2a horseradish peroxidase (HRP) conjugate detection antibody, diluted 2000–5000-fold, for 1 h at room temperature (Southern Biotech, Birmingham, AL; #1031-05, #1070-05, and #1080-05, respectively) followed by a wash then incubation with 25 μL of 1x 3,3′,5,5′-tetramethylbenzidine (TMB) substrate. The reaction was stopped using 25 μL of 1 N sulfuric acid, and absorbance was read at 450 nm using a Victor X4 plate reader (PerkinElmer, Waltham, MA). Endpoint titers were interpolated using a 4-parameter sigmoidal fit with a least squares regression and a cutoff value based on naïve serum. Samples which did not have a quantifiable titer were excluded from further analysis.

For quantification of RBD-specific IgA in BAL fluid, identical coating and blocking procedures were conducted. After washing, plates were incubated with BAL fluid at a final starting dilution of 5–25-fold followed by 11 2-fold dilutions and probed with 25 μL of goat anti-mouse IgA-HRP detection antibody (Southern Biotech #1040-05) diluted 1000-fold. The development procedure was performed as above, and absorbance was read at 450 nm. Titer calculations were performed as described above.

### 2.6 Bone marrow ELISpot

Antigen-specific antibody-secreting cells in murine bone marrow samples were quantified using an ELISpot assay*.* 96-well ELISpot plates (MilliporeSigma #MSIPS4W10) were coated with 100 μL of 2 μg/mL recombinant pre-fusion stabilized SARS-CoV-2 full-length wt Spike His Protein (R&D Systems, Minneapolis, MN, #10549-CV-100) in coating buffer (eBioscience #00004459) and incubated at 4°C overnight. Plates were then washed with PBST, followed by blocking with 250 μL complete Roswell Park Memorial Institute (RPMI) medium (RPMI + 1x GlutaMAX [ThermoFisher Cat# 35050079] + 10% [v/v] heat-inactivated fetal bovine serum [FBS]) for 2 h at ambient temperature and then washed again. 100 μL of single-cell suspensions of bone marrow were seeded at 1.0 × 10^6^ cells per well with five 3-fold serial dilutions (to ensure accurate counting) and incubated at 37°C with 5% CO_2_ for 3 h. Plates were washed, and 100 μL of HRP-conjugated anti-mouse IgG (H + L) (Southern Biotech #1031-05), diluted 1:1000, was added and incubated overnight at 4°C. The plates were washed and developed with 3-amino-9-ethylcarbazole (AEC) substrate kits (Vector Laboratories, Newark, CA) according to the manufacturer’s protocol. Plates were washed with excess deionized water to halt the reaction and dried in the dark. Colored spots were enumerated using an automated ELISpot reader (CTL Analyzer, Cellular Technology Limited, Cleveland, OH). Data were analyzed using ImmunoSpot software (Cellular Technology Limited). The resulting ELISpot data were confirmed to be lognormally distributed and were log_10_-transformed prior to plotting and analysis.

### 2.7 Splenocyte ELISpot

Cytokine-secreting mouse splenocytes were quantified by ELISpot assay. ELISpot plates were coated with 100 μL of anti-mouse IFN-y (BD Biosciences, Franklin Lakes, NJ, #551881), IL-5 (BD Biosciences #551880), or IL-17 (Invitrogen, Carlsbad, CA, #88-7371-88) capture antibodies at a dilution of 1:200 in coating buffer and incubated at 4°C overnight. Plates were washed with PBS, then blocked with 250 μL of complete RPMI medium for 2 h at ambient temperature, followed by another wash. 100 μL of splenocytes were plated at 2.0 × 10^5^ cells per well and stimulated with 100 μL of 1 μg/mL peptide pool of 1:1 (v:v) SARS-CoV-2 Subunit 1 (JPT Peptide Technologies, Berlin, Germany, #PM-WCPV-S-1) and SARS-CoV-2 Subunit 2 (JPT Peptide Technologies #PM-WCPV-S-2) at 37°C with 5% CO_2_ for 48 h. Plates were washed, then 100 μL of detection antibody (same as above), diluted 1:250, was added to the plates and incubated overnight at 4°C. After incubation, plates were washed, and 100 μL of avidin D (Av)-HRP (Invitrogen #50-112-3249), diluted 1:250, was added to the plates for 45 min at ambient temperature followed by a PBS wash. Plates were then developed with AEC substrate kits (Vector Laboratories) according to the manufacturer’s protocol. The plates were washed with excess deionized water to halt the reaction and dried in the dark. Colored spots were enumerated using an automated ELISpot reader (CTL Analyzer). Data were analyzed using ImmunoSpot software. The resulting ELISpot data were confirmed to be lognormally distributed and were log_10_-transformed prior to plotting and analysis.

### 2.8 Pseudovirus neutralization assay

SARS-CoV-2 pseudovirus neutralization assays were conducted on immunized mouse serum samples as previously described (Rice et al., 2022; Voigt et al., 2022). Briefly, lentiviral pseudoviruses expressing SARS-CoV-2 spike protein variants were prepared by co-transfecting HEK-293 cells (ATCC, Manassas, VA, #CRL-3216) seeded at 4.0 × 10^5^ cells/mL with a plasmid containing a lentiviral backbone expressing luciferase and ZsGreen (BEI Resources, Manassas, VA, #NR-52516), plasmids containing lentiviral helper genes (BEI Resources #NR52517, #NR-52518, and #NR-52519), a delta19 cytoplasmic tail truncated SARS-CoV-2 spike protein expression plasmid (Wuhan strain, Alpha B.1.1.7, and Beta B.1.351 spike variant plasmids were a gift from Jesse Bloom of Fred Hutchinson Cancer Center, Seattle, WA; Delta B.1.617.2 and Omicron B.1.1.529 variant plasmids were a gift from Thomas Peacock of Imperial College London, UK), and BioT transfection reagent (Bioland Scientific, Paramount, CA, #B0101). The transfection was incubated for 72 h at 37°C with 5% CO_2_. Pseudovirus stocks were harvested from the cell culture media (Gibco Dulbecco’s Modified Eagle Medium [DMEM] + GlutaMAX + 10% FBS), filtered through a 0.2-µm filter, and frozen at −80°C until use. Mouse serum samples were diluted 1:10 in media (Gibco DMEM + GlutaMAX + 10% FBS), then serially diluted 1:2 for 11 total dilutions and incubated for 1 h at 37°C with 5% CO_2_ and a mixture of 5 μg/mL polybrene (Sigma-Aldrich #TR-1003-G) and pseudovirus diluted to a titer that produces 1 × 10^8^ total integrated intensity units/mL in untreated cells. 100 μL of the serum-virus mix was then added in duplicate to human angiotensin-converting enzyme 2 (ACE-2) expressing HEK-293T cells (BEI Resources #NR-52511) seeded at 8 × 10^4^ cells per well on a 96-well plate. The plates were incubated at 37°C with 5% CO_2_ for 72 h. Plates were imaged on a high-content fluorescent imager (Molecular Devices ImageXpress Pico, San Jose, CA) for ZsGreen expression. Total integrated intensity units per well quantified using ImageXpress software (Molecular Devices) were used to calculate % pseudovirus inhibition in each well. Neutralization curves were fit with a four-parameter sigmoidal curve, which was used to calculate 50% inhibitory concentration dilution (IC_50_) values.

### 2.9 Fluorescence spectroscopy

RBD-adjuvant samples were prepared as described above and allowed to incubate at RT for 1 h before analysis. Fluorescence spectra were read on a Cary Eclipse Fluorescence Spectrometer (Agilent, Santa Clara, CA). Samples were loaded into a 10-mm path length low-volume quartz cuvette. Fluorescent spectra were read using a 600 V photomultiplier tube detector (excitation: 295 nm, emission: 310–380 nm). Before analysis, SE-containing samples were separated via ultracentrifugation (sampling from the denser aqueous phase) using an Optima MAX-XP (Beckman Coulter, Pasadena, CA) at 180,000 x g for 2 h. Alum-containing samples were left intact. *N =* 3 spectra were acquired per sample. Spectra were averaged and background corrected (using spectra of SE or Alum samples without RBD) before smoothing with a 20-point Savitzky-Golay filter. The center of spectral mass was determined via numerical integration using OriginPro 9.1 software (OriginLab, Northampton, MA).

### 2.10 Differential scanning fluorimetry (DSF)

RBD-adjuvant samples were prepared as described above and allowed to incubate at RT for 1 h before analysis. DSF measurements were performed using a Prometheus NT.48 (NanoTemper Technologies, München, Germany). Samples were loaded into high-sensitivity capillaries, sealed, and allowed to equilibrate to 15°C. Samples were heated from 15°C to 95°C at a rate of 0.2°C/min, while fluorescence measurements were acquired at 30% excitation power (excitation: 295 nm, emission: 330 nm and 350 nm). Melting temperatures were determined via NanoTemper software based on the 350:330 emission ratio as a function of temperature and reported as the average of three replicate measurements.

### 2.11 Statistical analyses

Adaptive immunity responses measured in vaccinated animals were log-transformed as indicated in figures. Normally distributed data were compared via a one- or two-way ANOVA followed by a Holm-Sidak’s correction for multiple comparisons, whereas non-normally distributed data were compared using the non-parametric Kruskal–Wallis test or Mann-Whitney test followed by Holm-Sidak’s or Dunn’s correction for multiple comparisons as indicated in figure legends. All statistical analyses were performed using GraphPad Prism 10.0.2 (San Diego, CA).

## 3 Results

### 3.1 Adjuvant immunogenicity screen

To determine the optimal adjuvant for use with the RBD subunit antigen, a library of adjuvants, including multiple agonists and formulations, was evaluated head-to-head in a prime-boost immunogenicity model using BALB/c mice that received two immunizations 3 weeks apart. Serum and BAL-based assays for each study (e.g., antibody titer, pseudovirus neutralization) were performed for all animals simultaneously using frozen serum and BAL samples, respectively. Assays relying on live cells (e.g., ELISpot) were performed at the time of tissue harvest. Experimental groups consisted of four agonists (GLA, 3M-052, CpG-1826, and dmLT) each formulated in SE or Alum, resulting in eleven total experimental groups including controls (Table 1). Serum samples were collected on Days 0, 21, and 42, and spleen, bone marrow, and BAL samples were collected on Day 42 (Supplementary Figure S1). Immunogenicity readouts were selected to measure the magnitude of the systemic humoral response in serum and the local humoral response at the lung surface as a metric of mucosal protection. IgG2a and IgG1 were used as metrics of Th1 and Th2 immunity, respectively, and their ratio was used to quantify the immune phenotype induced by each vaccine (Bal et al., 2011; Schwenk et al., 2014).

**TABLE 1:** General dosing scheme for all studies. Doses were given per animal per vaccination. All vaccines were dosed intramuscularly in 100 μL total volume, split into two 50 μL boluses given in the rear biceps femoris muscle.

Group #	Experimental group	RBD antigen (μg)	Formulation	Agonist
1	Antigen Control	7	-	-
2	Alum	7	Alum (100 μg)	-
3	GLA-Alum	7	Alum (100 μg)	GLA (5 μg)
4	3M-052-Alum	7	Alum (100 μg)	3M-052 (2 μg)
5	CpG-Alum	7	Alum (100 μg)	CpG (20 μg)
6	dmLT-Alum	7	Alum (100 μg)	dmLT (0.3 μg)
7	SE	7	SE (2% v/v)	-
8	GLA-SE	7	SE (2% v/v)	GLA (5 μg)
9	3M-052-SE	7	SE (2% v/v)	3M-052 (2 μg)
10	CpG-SE	7	SE (2% v/v)	CpG (20 μg)
11	dmLT-SE	7	SE (2% v/v)	dmLT (0.3 μg)

**FIGURE 1:**
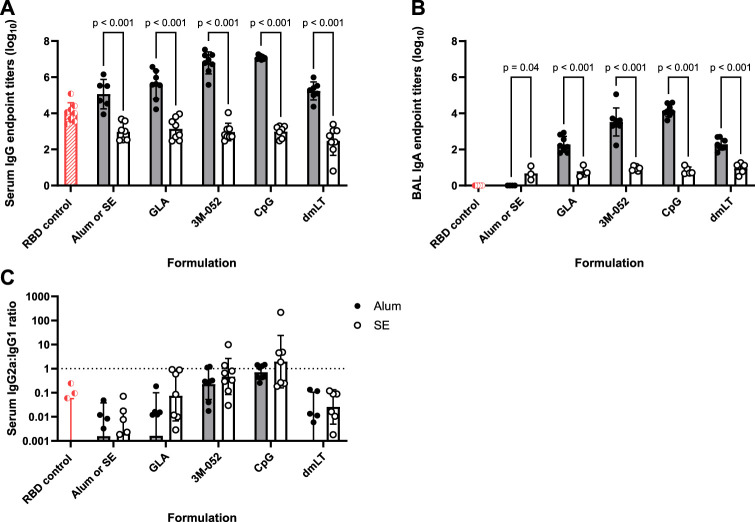
Formulation choice drives humoral response. Data collected from *n* = 8 (4M:4F) animals on Day 42 after being vaccinated twice intramuscularly (Day 0 and Day 21) with RBD in combination with the indicated receptor agonist formulated with either Alum or SE. RBD control data (red striped) were collected from animals who were vaccinated twice with RBD, without the addition of an adjuvant. The study was divided in half and vaccinations/harvests were staggered 1 week apart to reduce operator burden. Assays presented here were performed for all animals simultaneously using frozen serum and BAL samples. Animals in the Alum-containing groups and the RBD control group were vaccinated and harvested on different days than those in the SE-containing groups. **(A)** Serum titer of total anti-RBD IgG, **(B)** BAL titer of anti-RBD IgA, and **(C)** serum ratio of exponentiated anti-RBD IgG2a/IgG1 titers. **(A, B)** Statistical significance was determined via repeated *t*-tests followed by a Holm-Sidak’s correction for multiple comparisons, fixing the family-wide error rate to 0.05. Horizontal bars represent the mean ± SD of log-normalized data. **(C)** Statistical significance was determined via multiple non-parametric Mann-Whitney tests followed by Holm-Sidak’s correction for multiple comparisons, fixing the family-wide error rate to 0.05. Horizontal bars represent the geometric mean ± geometric SD.

Total serum anti-RBD endpoint IgG titers measured from Day 42 serum (Figure 1A) showed that, in general, Alum formulations led to significantly greater production of serum IgG compared to SE-based formulations regardless of the agonist used (*p <* 0.001 for all comparisons). Both CpG and 3M-052 on Alum led to a statistically significant increase in serum IgG titers of 2.183 (*p <* 0.001) and 3.560 (*p <* 0.001) logs, respectively, compared to Alum formulated without additional agonists (Supplementary Figures S2A, B), whereas all SE-agonist formulations did not statistically increase serum IgG titers compared to SE without agonists (*p* > 0.2 for all comparisons, Supplementary Figures S3A, B). BAL anti-RBD IgA was measured as a metric of humoral protection at the point of infection in the lower respiratory tract. Agonist-containing Alum formulations led to significantly higher BAL IgA titers compared to the same agonist formulated with SE (Figure 1B), increasing BAL IgA titers by 1.328 logs at minimum and by up to 3.327 logs in the case of CpG formulated on Alum (*p <* 0.001 for all comparisons). The ratio of serum IgG2a to IgG1 titer was used as a correlate for the relative balance of the Th1- and Th2-type response, expressed as the quotient of the exponentiated log titer values. No significant differences in the IgG2a/IgG1 ratio were observed between agonist groups formulated with Alum and those formulated with SE (*p* > 0.65 for all comparisons) (Figure 1C). 3M-052 and CpG significantly increased the serum IgG2a/IgG1 ratio when formulated in either Alum or SE by 9.0- to 17.3-fold or 132.8- to 2586.9-fold compared to either Alum or SE formulated without an agonist, respectively (*p <* 0.02 for all comparisons) (Supplementary Figures S2C, S3C). CpG-Alum and 3M-052-SE produced the most balanced IgG2a/IgG1 ratios (0.86 ± 0.50 and 1.69 ± 3.35, respectively), while the CpG-SE adjuvant formulation produced the only geometric mean IgG2a/IgG1 ratio that was greater than unity (1.953 ± 12.1).

**FIGURE 2:**
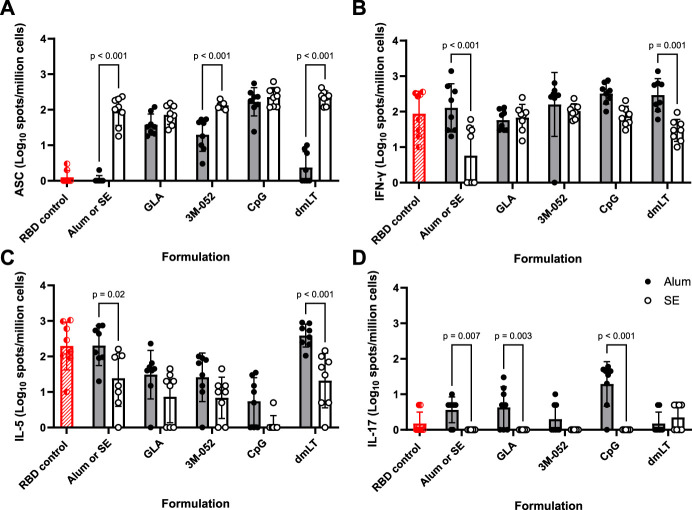
Formulation choice affects immune phenotype. ELISpot data collected from *n* = 8 (4M:4F) animals on Day 42 after being vaccinated twice intramuscularly (Day 0 and Day 21) with RBD in combination with the indicated Alum- or SE-formulated receptor agonist. RBD control data (red striped) were collected from animals who were vaccinated twice with RBD without the addition of an adjuvant. Study groups were divided in half and vaccinations/harvests were staggered 1 week apart to reduce operator burden. Assays presented here were performed at the time of tissue harvest. Animals in the Alum-containing groups and the RBD control group were vaccinated and harvested on different days than those in the SE-containing groups. **(A)** Bone marrow-derived anti-full-length-wt-spike IgG antibody-secreting cells (ASC) ELISpot. T cell ELISpot measurement of splenocytes secreting **(B)** IFN-γ, **(C)** IL-5, or **(D)** IL-17 upon re-stimulation with a SARS-CoV-2 peptide pool. Horizontal bars represent the mean ± SD of the log-transformed data. Statistical significance was determined via two-way ANOVA followed by Holm-Sidak’s correction for multiple comparisons.

Anti-full-length-wt-spike-binding IgG ELISpot assays were performed on bone marrow-derived cells as a metric of humoral immune memory. SE formulated with 3M-052, dmLT, or without an agonist significantly increased the bone marrow anti-full-length-wt-spike IgG response by 0.8, 2.0, and 1.9 logs, respectively (*p <* 0.001 for all comparisons), compared to the equivalent Alum formulations (Figure 2A). GLA, 3M-052, and CpG formulated on Alum all significantly increased the bone marrow anti-full-length-wt-spike IgG response in comparison to Alum without an additional agonist (*p <* 0.001 for all comparisons) (Supplementary Figure S4A). CpG and dmLT formulated with SE significantly increased the bone marrow anti-full-length-wt-spike IgG response in comparison to SE without an additional agonist (*p* = 0.02 for both comparisons) (Supplementary Figure S5A).

Splenocyte cytokine ELISpots were used as a metric of adaptive cellular immune memory. The pro-inflammatory Th1 IFN-γ ELISpot response induced by dmLT formulated on Alum or Alum without an additional agonist was significantly higher than the corresponding SE responses by 1.0 and 1.3 logs, respectively (*p* < 0.001 for both comparisons) (Figure 2B). There was no significant effect of agonist choice on the splenocyte IFN-γ ELISpot response in Alum-containing groups (*p* > 0.5 for all comparisons) (Supplementary Figure S4B). GLA, 3M-052, CpG, and dmLT all significantly amplified the splenocyte IFN-γ response when compared to SE without an additional agonist (*p* < 0.004 for all comparisons) (Supplementary Figure S5B).

IL-5 splenocyte ELISpot responses were measured as indicators of Th2-type responses. dmLT formulated on SE and SE without an additional agonist significantly decreased the IL-5 ELISpot response by 1.3 and 0.9 logs, respectively, in comparison to the corresponding Alum formulation (*p* < 0.02 for both comparisons) (Figure 2C). GLA, 3M-052, and CpG formulated with Alum significantly decreased the splenocyte IL-5 ELISpot response (Supplementary Figure S4C) in comparison to Alum without an additional agonist (*p* < 0.02 for all comparisons). CpG-SE significantly reduced the splenocyte IL-5 ELISpot response when compared to SE without an additional agonist (*p* < 0.001) (Supplementary Figure S5C).

IL-17 splenocyte ELISpot responses were measured as indicators of Th17-type responses. GLA and CpG on Alum and Alum without an additional agonist significantly increased the IL-17 response by 0.6, 1.3, and 0.6 logs, respectively, in comparison to the corresponding SE formulation (*p* < 0.003 for all comparisons) (Figure 2D). CpG-Alum significantly increased the splenocyte IL-17 ELISpot response in comparison to Alum without an additional agonist (*p* = 0.02) (Supplementary Figure S4D). The splenocyte IL-17 ELISpot responses of SE-containing groups were uniformly below our limit of detection with the exception of dmLT-SE, which significantly increased the splenocyte IL-17 ELISpot response from baseline (*p* < 0.001) (Supplementary Figure S5D).

### 3.2 Lead adjuvant selection via aggregate desirability score

Lead adjuvant candidates for use with the RBD antigen were chosen from our immunogenicity screen using a desirability index approach. A desirability index is a means to rank multiple groups across multiple parameters simultaneously by applying a weighting scheme to outputs and aggregating results into a single score for each group (Costa et al., 2011; Abhyankar et al., 2021). The best-performing groups can then be selected by the highest scores. Briefly, for each output parameter, a weight (from 1 to 5) was assigned, with higher-weighted parameters being more influential on the overall score. Next, each parameter was assigned to either be maximized, rewarding high relative values, or minimized, rewarding low relative values. The weighting scheme used in this study can be found in Table 2. Weights were chosen to emphasize known correlates of clinical responses: pseudovirus neutralization (Supplementary Figure S7), bone marrow-derived antibody-secreting cells, and serum antibody titer while minimizing the pro-Th2 marker IL-5 (Lederer et al., 2020; McMahan et al., 2021; Gilbert et al., 2022). A detailed description of this method can be found in the Supplementary Material.

**TABLE 2:** Desirability index weighting scheme. Weights were assigned from 1 to 5, from least to most important/desirable. Further method details are available in the Supplementary Methods.

Parameter	Unit	Weight	Optimization goal
Pseudovirus Neutralizing Titer	IC_50_ (Log_10_)	5	Maximize
Bone Marrow IgG ELISpot	Spots/Million Cells	4	Maximize
Serum anti-RBD IgG: Day 42	Titer (Log_10_)	3	Maximize
Serum IgG2a/IgG1 Ratio: Day 42	Ratio of exponentiated titers	3	Maximize
IFN-γ ELISpot	Spots/Million Cells	2	Maximize
BAL anti-RBD IgA	Titer (Log_10_)	2	Maximize
IL-17 ELISpot	Spots/Million Cells	1	Maximize
Serum anti-RBD IgG: Day 21	Titer (Log_10_)	1	Maximize
Serum IgG2a/IgG1 Ratio: Day 21	Ratio of exponentiated titers	1	Maximize
IL-5 ELISpot	Spots/Million Cells	2	Minimize

**FIGURE 3:**
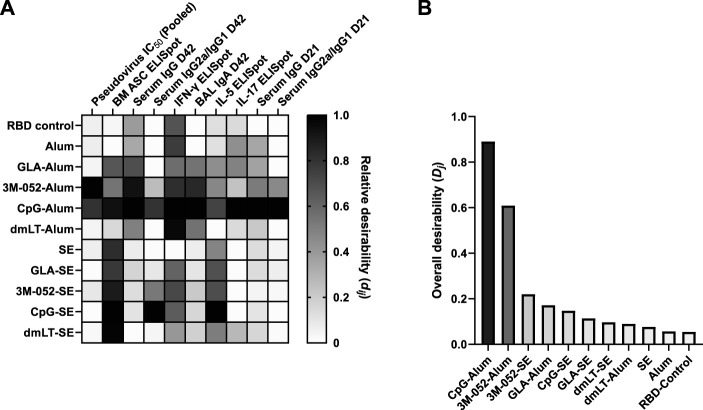
Identification of the most desirable adjuvant formulation for use with the SARS-CoV-2 RBD. CpG and 3M-052 on Alum were the highest-scoring adjuvant formulations based on our desirability index weighting (Table 2). Desirability index results were calculated using formulas described in the Supplementary Methods. **(A)** Heatmap of individual desirability index scores (*d*_*ij*_), for the *j*th group and *i*th response. Columns are ordered left-to-right by decreasing weight (see Table 2). Scores are normalized within each response variable. D = Day, BM = bone marrow. **(B)** Weighted, aggregate desirability scores (*D*_*j*_) per group (*j*), ordered left-to-right from highest to lowest score. RBD control represents RBD without the addition of an adjuvant. Animals in the Alum-containing groups and the RBD control group were vaccinated and harvested on different days than those in the SE-containing groups.

A heat map depicting the relative values of the desirability score ( $\left.d_{ij}\right)$ for each group and parameter is shown in Figure 3A, and aggregate scores are shown in Figure 3B. Day 21 serum IgG and IgG2a/IgG1 and pooled pseudovirus neutralization data are shown in Supplementary Figures S6, S7, respectively. Aggregate desirability scores (*D*_*j*_) are normalized to a range of 0–1, with higher results being more desirable based on the pre-defined weighting criteria. The adjuvant formulations with the highest aggregate desirability scores were CpG-Alum (*D*_CpG-Alum_ *=* 0.891) and 3M-052-Alum (*D*_3M-052-Alum_ *=* 0.609) (Figure 3B). The next highest-scoring adjuvant formulations were 3M-052-SE (*D*_3M-052-SE_ *=* 0.220) followed by GLA-Alum (*D*_GLA-Alum_ *=* 0.172), CpG-SE (*D*_CpG-SE_ = 0.147), GLA-SE (*D*_GLA-SE_ = 0.114), dmLT-SE (*D*_dmLT-SE_ = 0.097), and dmLT-Alum (*D*_dmLT-Alum_ = 0.090). All agonist-containing adjuvant formulations outperformed the agonist-free SE (*D*_SE_ = 0.076) and Alum (*D*_Alum_ = 0.056) formulations, which both outperformed the unadjuvanted RBD control (*D*_RBD_ = 0.055), the lowest performing formulation (Figure 3B). Based on these results, the CpG-Alum and 3M-052-Alum adjuvant formulations were selected for further study with a bivalent RBD antigen approach.

### 3.3 Effect of formulation on physical properties of the RBD antigen

**FIGURE 4:**
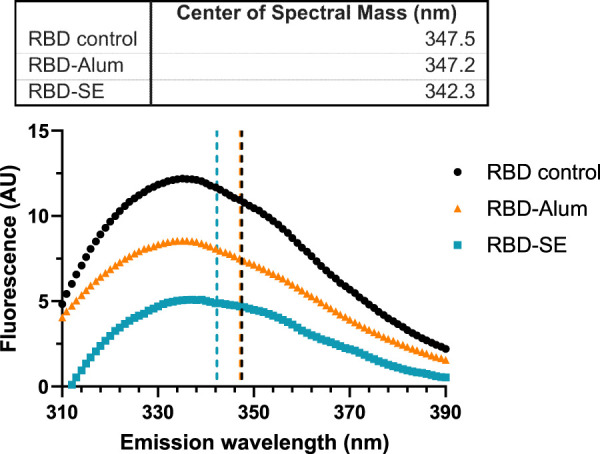
Formulation with SE causes a blue shift in fluorescence spectra. Data are representative of the average of three replicate measurements after applying a Savitzky-Golay filter to reduce signal noise. Samples were excited at 295 nm, and emissions were read between 310 and 450 nm (only 310–390 nm shown). Centers of spectral mass were determined for each formulation and are indicated by the corresponding dashed lines. Statistically significant differences between distributions were determined via multiple Kolmogorov–Smirnov tests, followed by Holm-Sidak’s correction for multiple comparisons (RBD vs. RBD-Alum: *p* = 1. RBD vs. RBD-SE: *p* < 0.001. RBD-SE vs. RBD-Alum: *p* < 0.001).

To better understand how the vaccine composition influenced immunogenicity, we explored the effect of the formulation components on the tertiary structure of the RBD antigen. Fluorescence spectroscopy was used to measure conformational shifts in the antigen via its intrinsic tryptophan residues whose maximum emission wavelength changes based on the local chemical environment. RBD203-N1 has two tryptophan residues, neither of which are in the ACE-2 receptor binding motif (Chen et al., 2022). RBD samples were formulated with either SE, Alum, or were left neat at concentrations equivalent to previous immunogenicity studies (Table 1). However, no additional agonists were included to limit confounding spectra. The center of spectral mass for the RBD control (Figure 4) suggests that its tryptophan residues are already solvent-exposed. A ∼5 nm blue shift in the center of mass of the fluorescence emission spectra of the SE-formulated RBD (Figure 4) suggests that one or more RBD tryptophan residues entered a less-polar local environment (Chapeaurouge et al., 2002). The statistical significance of this shift was confirmed via multiple two-sample Kolmogorov–Smirnov tests, followed by Holm-Sidak’s correction for multiple comparisons (RBD vs. RBD-Alum: *p =* 1. RBD vs. RBD-SE: *p <* 0.001. RBD-SE vs. RBD-Alum: *p <* 0.001). Because the SE was removed prior to analysis, it is likely that the observed shift in spectra center of mass of the SE-formulated RBD was due to a conformational change in the antigen, which was not observed in the Alum-formulated RBD.

**FIGURE 5:**
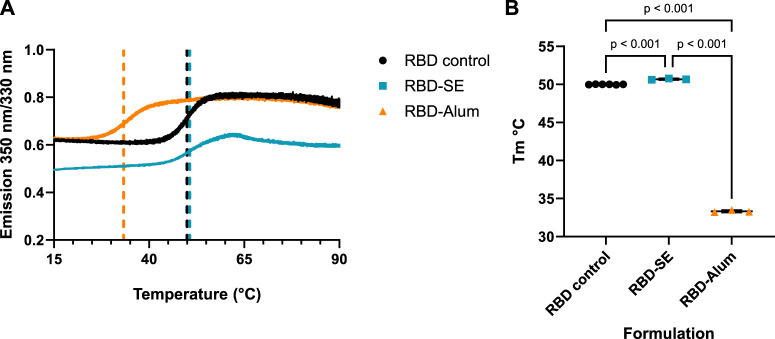
Formulation with Alum lowers RBD melting temperature (Tm). **(A)** Melting curves of RBD samples in different formulations. Samples were heated to 95°C at a rate of 0.2°C/min. Data are averaged from 3 to 6 replicate measurements. Dashed lines indicate the Tm of the corresponding formulation. **(B)** Calculated Tm by formulation. Tm was calculated as the inflection point of the second derivative of the 350/330 nm vs. temperature curve. Horizontal bars represent the mean ±1 SD. Note: standard deviations are plotted but are not visible. Statistical significance was determined via one-way ANOVA followed by a Holm-Sidak’s correction for multiple comparisons.

Differences in formulated RBD melting temperature (Tm), also an assessment of protein conformation, were measured via differential scanning fluorimetry (DSF). The initial (i.e., low temperature) 350:330 emission ratio measured for each formulation was consistent with the center of spectral mass values in Figure 4 with a similar ratio for the RBD control and RBD-Alum and a lower ratio for the RBD-SE, indicating a more hydrophobic environment for the tryptophan residues in the RBD-SE formulation. Formulation with both SE and Alum caused statistically significant shifts from the native RBD Tm of 49.99 °C (Figure 5), suggesting an impact on the antigen’s conformation in both cases. Formulation with SE increased the Tm by 0.70°C–50.69°C (*p <* 0.001), and formulation with Alum decreased the Tm by 16.66°C–33.33°C (*p <* 0.001). Our immunology readouts demonstrate that the Alum-containing formulations maintained their immunogenicity effectively, but the decreased Tm may indicate a loss of long-term stability for the RBD antigen formulated with Alum. This further suggests that formulation with either SE or Alum led to distinct changes in the structure of the RBD antigen, at both ambient and elevated temperatures.

RBD physical stability after mixing was determined via SDS-PAGE (see Supplementary Material). Complete recovery of the RBD was observed from all SE-containing groups, and complete Alum adsorption was observed in all Alum-containing groups except for dmLT-Alum, where there appears to be 4%–6% (w/w) unbound protein (Supplementary Figure S8). This demonstrates that there was no apparent chemical degradation of the RBD antigen during the formulation process, which would be indicated by either multiple bands in the SE-formulated samples or additional bands in the Alum-formulated samples. The origin of the visible bands in the dmLT-Alum samples is unknown. Although unverified, these may be RBDs displaced from the Alum.

### 3.4 Effect of antigen and adjuvant on cross-variant protection

The emergence of additional SARS-CoV-2 variants of interest beyond the original Wuhan (wt) strain prompted the investigation of bivalent antigens targeting different variants of interest as a means to enhance the breadth of the immune response. At the time this study was performed, the Delta variant was a major concern, leading us to investigate the use of wt and Delta variant RBDs, individually or in a bivalent combination. The same study outline, dosing schedule, and assays were performed as before, using either CpG-Alum or 3M-052-Alum as adjuvant formulations (Table 1). All animals received either 7 μg of the wt RBD, 7 μg of the Delta RBD, or 3.5 μg of both the wt and Delta RBD (7 μg total).

**FIGURE 6:**
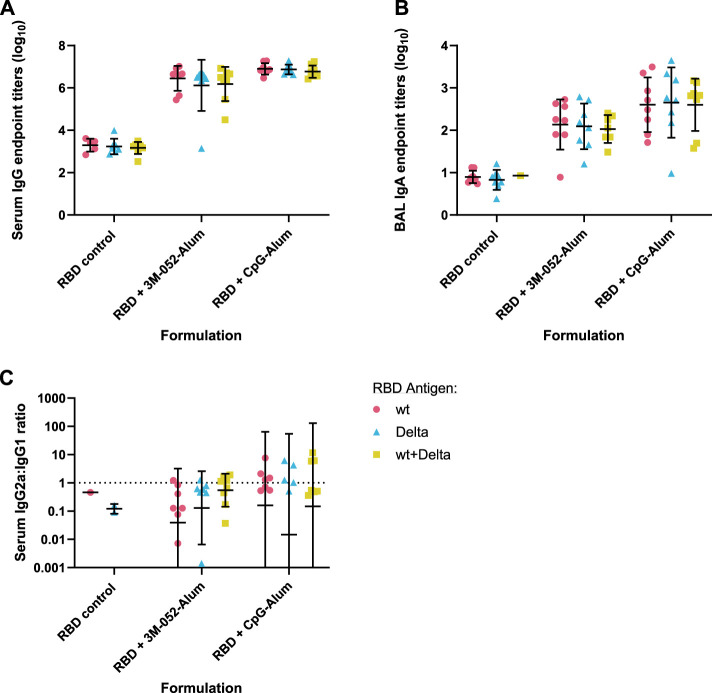
Antigen bivalency does not affect overall mouse wt RBD-binding antibody titers. Data were collected from *n* = 8 (4M:4F) animals vaccinated twice intramuscularly with the indicated adjuvant and antigen combination. Animals received 7 μg of either wt RBD or Delta RBD, or 3.5 μg of both. RBD control data were collected from animals who were vaccinated with the indicated RBD variant(s) without the addition of an adjuvant. Serum and BAL samples were collected 42 days post-prime. Study groups were divided evenly in half and vaccinations/harvests were staggered 1 week apart to reduce operator burden. Assays presented here were performed for all animals simultaneously using frozen serum and BAL samples. **(A)** Serum titer of total anti-wt-RBD-reactive IgG, **(B)** BAL titer of anti-wt-RBD IgA, and **(C)** serum ratio of exponentiated anti-wt-RBD IgG2a/IgG1 titers. **(A, B)** Horizontal bars represent the mean ±1 SD of log-normalized data. **(C)** Horizontal bars represent the geometric mean ± geometric SD. **(A–C)** Statistical significance was determined by two-way ANOVA with a full effects model, followed by Holm-Sidak’s correction for multiple comparisons, fixing the family-wide error rate at 0.05.

Readouts for serum anti-wt RBD IgG (Figure 6A), BAL wash anti-wt RBD IgA (Figure 6B), and serum anti-wt RBD IgG2a/IgG1 ratio (Figure 6C) showed no significant differences between antigen groups (*p* > 0.34 for all comparisons). The choice of adjuvant was found to be the only statistically significant source of variation (*p <* 0.001 in all cases via two-way ANOVA).

**FIGURE 7:**
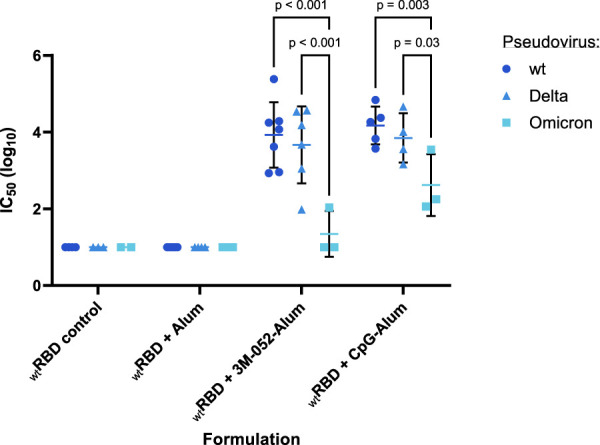
Serum-neutralizing antibody titer induction depends on both adjuvant and antigen choice. Sera from *n* = 4-6 animals vaccinated twice with the wt RBD antigen formulated with the listed adjuvant (prime Day 0, boost Day 21) were tested in a pseudovirus neutralization assay against a Wuhan (wt), a Delta (B.1.617.2), and an Omicron (B.1.1.529) strain pseudovirus. RBD control data were collected from animals who were vaccinated with RBD without the addition of an adjuvant. Serum samples were harvested from animals 42 days post-prime. Study groups were divided evenly in half and vaccinations/harvests were staggered 1 week apart to reduce operator burden. Assays presented here were performed for all animals simultaneously using frozen serum samples. Horizontal bars represent the mean ±1 SD of log-normalized data. Statistical significance was determined via a full model two-way ANOVA, followed by a Holm-Sidak’s correction for multiple column-wise comparisons, limiting the family-wide error rate to 0.05.

SARS-CoV-2 wt (Wuhan), Delta (B.1.617.2), or Omicron (B.1.1.529) strain neutralizing titers of vaccinated mouse sera were quantified using established *in vitro* SARS-CoV-2 variant spike protein-expressing lentiviral pseudovirus neutralization assays (Rice et al., 2022; Voigt et al., 2022). The neutralizing IC_50_ of serum from animals that had received the wt RBD vaccine antigen either unadjuvanted or formulated with Alum, 3M-052-Alum, or CpG-Alum were thus measured. None of the animals that received the unadjuvanted wt RBD or the wt RBD formulated on Alum without an additional agonist displayed a serum-neutralizing IC_50_ value above our limit of detection against any of the three tested SARS-CoV-2 variants (Figure 7). There was no significant difference in serum IC_50_ against the wt or Delta variant pseudoviruses from mice that received the wt RBD adjuvanted with either 3M-052-Alum (*p =* 0.44) or CpG-Alum (*p =* 0.43). The mean serum virus neutralizing IC_50_ response from the adjuvanted wt RBD groups against the Omicron variant pseudovirus was significantly lower than the responses against the wt or Delta pseudovirus by about 2 logs for animals vaccinated with wt RBD formulated with 3M-052-Alum (*p <* 0.001 for both comparisons), and by about 1 log for animals vaccinated with wt RBD formulated with CpG-Alum (*p <* 0.02 for both comparisons).

**FIGURE 8:**
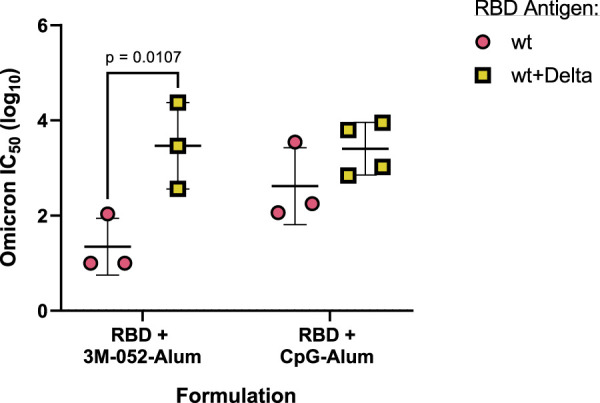
Broadening of neutralizing response is dependent on both antigen and adjuvant composition. Animals received the indicated antigen-adjuvant combinations on Days 0 and 21 (*n* = 4–6), and serum samples were taken 42 days post-prime. All animals received either 7 μg of wt RBD or 3.5 μg of both wt and Delta RBD. IC_50_ neutralization values of serum from mice that received the indicated antigen formulated with the listed adjuvant were tested in a pseudovirus neutralization assay against an Omicron strain (B.1.1.529) pseudovirus. Study groups were divided evenly in half and vaccinations/harvests were staggered 1 week apart to reduce operator burden. Assays presented here were performed for all animals simultaneously using frozen serum samples. Horizontal bars represent the mean ±1 SD of log-normalized data. Statistical significance was determined via multiple *t*-tests, followed by a Holm-Sidak’s correction for multiple comparisons, limiting the family-wide error rate to 0.05. Note: wt RBD data are also presented in Figure 7.

To understand the effect of bivalency on neutralization against an Omicron variant (B.1.1.529) pseudovirus, we compared the serum-neutralizing IC_50_ of mice that had received either only the wt RBD or both the wt and the Delta RBDs, formulated with either 3M-052-Alum or CpG-Alum. Vaccination with the bivalent wt + Delta RBD antigens significantly increased the Omicron strain neutralizing IC_50_ of mice that had received the 3M-052-Alum adjuvanted vaccine by 2.12 logs (*p =* 0.0107) but did not significantly improve the neutralizing IC_50_ of mice that had received the CpG-Alum adjuvanted vaccine (*p =* 0.333) despite a mean increase of 0.79 logs (Figure 8). This suggests that there was a degree of response broadening with the bivalent RBD antigen formulated with 3M-052-Alum, but the strength of the CpG-Alum adjuvanted wt RBD was strong enough against the Omicron pseudovirus that no significant difference was detected compared to the bivalent antigen.

Cellular responses in the bone marrow and splenocyte samples were measured via ELISpot assays as above. No significant differences between antigen groups were detected in anti-full-length-wt-spike IgG bone marrow ELISpot (Supplementary Figure S9A) or splenocyte IFN-γ ELISpot assays (Supplementary Figure S9B) (*p* > 0.3 for all comparisons). The unadjuvanted bivalent wt + Delta RBD induced fewer IL-5-secreting splenocytes than the unadjuvanted monovalent wt RBD group (Supplementary Figure S9C), and the monovalent wt RBD group adjuvanted with CpG-Alum induced more IL-17-secreting splenocytes than the corresponding monovalent Delta RBD group (Supplementary Figure S9D) (*p* < 0.01 for both comparisons). For other adjuvant groups, antigen choice did not significantly impact IL-5 or IL-17 ELISpot responses (*p* > 0.06 for all comparisons).

## 4 Discussion

In this study, we explored the use of the RBD203-N1 antigen derived from SARS-CoV-2 in combination with different immunogenic receptor agonists and formulations and explored the effect of antigen bivalency in BALB/c mice. The results summarized here demonstrate that the choice of adjuvant and formulation has a measurable effect on the immune phenotype and physical properties of an antigen that has demonstrated clinical value.

We observed that Alum-based adjuvant systems outperformed SE-based systems in terms of serum IgG titer and BAL IgA titer. Indeed, the formulation of an agonist in SE did not increase the magnitude of the antigen-specific IgG (Figure 1A) or IgA responses (Figure 1B) compared to SE without the addition of an agonist. This is notable given the large amount of preclinical and clinical data demonstrating the immunogenicity and efficacy of vaccines containing SE or emulsion-based adjuvants with subunit antigens (Fox and Haensler, 2013; Facciolà et al., 2022). Interestingly, previous studies have shown that 3M-052-SE is a very potent adjuvant when formulated with full-length SARS-CoV-2 Spike protein (Garrido et al., 2021; Milligan et al., 2023), demonstrating protection for at least 1 year following immunization in infant rhesus macaques, suggesting that the RBD antigen is non-optimal with SE-based formulations potentially due to its truncated nature as opposed to a sequence-related issue. Compared to aluminum-based adjuvants that electrostatically bind to protein antigens, emulsion-based adjuvants are generally thought to not interact directly with protein antigens but instead promote a local immune environment that facilitates lymphatic drainage (HogenEsch et al., 2018; Facciolà et al., 2022; Laera et al., 2023). Other studies have shown that the direct interactions between Alum and antigens are often necessary to elicit an effective immune response, or they can diminish the immunogenicity through deleterious interactions (Fox et al., 2016; HogenEsch et al., 2018).

Our results agree with previous findings that the RBD antigen tends to be more immunogenic when using pattern recognition receptor ligands formulated with aluminum-based adjuvants than when using emulsion-based adjuvants (Nanishi et al., 2022). These results suggest that there is likely some inherent biophysical interaction between the Alum particle and the RBD adsorbed onto its surface that mediates the immunogenicity of the RBD. Our results show that formulation with SE causes a statistically significant blue shift in the fluorescent spectra of the RBD antigen (Figure 4) (Chapeaurouge et al., 2002; Ramella et al., 2011; Dos Santos Rodrigues et al., 2023). Generally, this would be thought of as a stabilizing shift, and in our measurement of RBD Tm, we see that formulation with SE increases the Tm by 0.7°C. We also observe that formulation with Alum decreases the Tm of the RBD by nearly 17°C, even though the results of our desirability index model show that, in general, pattern recognition receptor ligands with Alum-based formulations improve the immunogenicity of the RBD antigen compared to equivalent SE-based formulations (Figure 3). While it is a highly non-linear measurement, we observe that the addition of CpG to the RBD-Alum vaccine increases our desirability metric by more than 15-fold, while the addition of CpG to the RBD-SE vaccine increases our desirability metric by less than 2-fold. A potential interpretation of these results is that formulation of RBD with Alum enhances the adjuvanting ability of these agonists. These findings highlight the importance of empirically assessing antigen-adjuvant compatibility in the development and evaluation of vaccine candidates.

Our results with a bivalent RBD antigen demonstrate that the combination of two related antigens can sometimes improve the neutralization capacity against a more distant third strain. We show that the 3M-052-Alum adjuvant in combination with both wt RBD and Delta RBD significantly improves the neutralizing titer against an Omicron variant pseudovirus in comparison to the monovalent wt RBD antigen formulated in the same way (Figure 8). This broadening effect was adjuvant dependent, as the neutralizing response of the CpG-Alum adjuvant with the monovalent wt RBD antigen was statistically equivalent to that of the bivalent antigen vaccine. This agrees with previous results looking at response broadening via multivalent RBD antigens, including other studies showing that including separate RBDs from two distinct variants can improve the neutralization capacity against a more distant third variant (Nanishi et al., 2022; Xu et al., 2023). These results further confirm the importance of not only appropriate adjuvant choice but also appropriate antigen choice in bivalent vaccine development. The molecular and immunological mechanisms underlying this apparent epitope-broadening phenomenon merit further investigation.

Previous data have shown that the immunogenicity of the RBD antigen in combination with several common receptor agonists (including CpG) is further improved by the addition of Alum (Nanishi et al., 2022). Conversely, multiple studies have shown that oil-in-water emulsions, similar to SE, without additional agonists are non-optimal adjuvants for use with RBD-based vaccines (Chen et al., 2020; Nanishi et al., 2022). There has also been additional precedent showing that often the adjuvanting ability of GLA and 3M-052 are benefited by formulation with Alum or SE (Anderson et al., 2010; Fox et al., 2016). Thus, for the purposes of this study, receptor agonists formulated without Alum or SE were only explored in a limited fashion. Initial studies of the RBD antigen adjuvanted with 3M-052 or CpG formulated without Alum (Supplementary Figure S10), but instead given as an aqueous formulation (AF), showed that Alum was necessary to generate robust serum anti-RBD IgG (Supplementary Figure S10A), BAL anti-RBD IgA (Supplementary Figure S10B), and bone marrow- resident anti-full-length-wt-spike IgG- secreting cells (Supplementary Figure S10C). For these reasons, this study was focused on understanding the differences between agonists formulated on Alum or SE as opposed to agonists formulated directly with the RBD antigen. However, this does mean that this study is unable to decouple the signal of the agonist from the signal of the Alum or SE, and it is unable to determine if the Alum or SE is beneficial in combination with a given agonist in the context of the RBD antigen.

There are several limitations to more definitive interpretations of these results. In the initial screening study (section 3.1–3.2), the Alum- and SE-based vaccines were tested on separate days to reduce operator burden, meaning that our results are potentially partially confounded by unaccounted-for day-to-day variation. The animal study presented in Section 3.4 was evenly divided based on group and animal sex, so no day-to-day bias is expected. Additionally, due to product-specific manufacturing requirements, the compositions of 3M-052-SE and the other SE products are slightly different: Most SE formulations use DMPC as an emulsifying agent, whereas 3M-052-SE uses egg PC (additionally, 3M-052-SE contains alpha-tocopherol as an antioxidant). Next, while serum pseudovirus neutralization is a useful measurement, it is not necessarily representative of true protection from viral challenge. We also cannot rule out the possibility that the current observations are unique to BALB/c or other inbred mouse strains but are not generally translatable to other model systems (Zeng et al., 2016). It is also known that there are species-dependent differences in the expression of and structure of TLRs and other pathogen recognition receptors between mice and humans (Kaisho and Akira, 2006; Kawai and Akira, 2009). Notably, the CpG-1826 used in this study is designed to bind tightly to the murine TLR9 but is known to bind less effectively to the human TLR9 than the commercially available CpG-1018, even though they are in the same ODN class (Campbell, 2017). In general, mice are known to express TLR9 on both macrophages and dendritic cells (DCs), whereas humans primarily express TLR9 on B cells and the plasmacytoid DC subset (Bode et al., 2011). Furthermore, 3M-052 is an agonist of both human TLR7 and TLR8, both of which are present in monocyte populations. Mouse TLR8 is homologous to the human gene; however, its relevancy in murine immunity is controversial, and it is likely that murine TLR7 drives the observed response to 3M-052 (Zeng et al., 2016; Lee et al., 2022). Finally, our desirability weighting scheme is a potential source of bias. Weights were chosen while all investigators were blinded to immunological readouts; however, selection of different weights would change the desirability ranking of some of the tested adjuvant formulations.

In conclusion, this study demonstrates that the immune phenotype of a subunit RBD vaccine against SARS-CoV-2 can be tuned by optimizing the adjuvant (formulation and agonist) and antigen choice. We believe these results could help inform the development of next-generation subunit vaccines against SARS-CoV-2 and other pandemic pathogens.

## Data Availability

The raw data supporting the conclusion of this article will be made available by the authors, without undue reservation.
