# Supplementary material for: Choice of adjuvant and antigen composition alters the immunogenic profile of a SARS-CoV-2 subunit vaccine
Source: Front Drug Deliv. 2024 Feb 7;4:1342518. doi: 10.3389/fddev.2024.1342518 (PMC12363249; doi:10.3389/fddev.2024.1342518)
Supplement: Supplementary file 1 [file DataSheet1.PDF]

# *Supplementary Material*

## 1 SUPPLEMENTARY METHODS

### Desirability Index calculations

Replicate data from each group were condensed into a descriptive statistic, using the geometric mean for all samples. Each group was then assigned a score for each parameter via Eqn. 1 to maximize a parameter or Eqn. 2 to minimize a parameter.

Where $d_{ij}$ is the desirability of the $i^{\text{th}}$ parameter of group $j$, and $\mu_{ij}$ is the descriptive statistic of the $i^{\text{th}}$ parameter of group $j$, $UB_i$ (upper bound of parameter $i$) and $LB_i$ (lower bound of parameter $i$) were calculated using Eqn. 3 and Eqn. 4, respectively. Where $\mu_{i:}$ is a vector containing all descriptive statistics for parameter $i$. Once all $d_{ij}$ were known, the aggregate desirability of group $j$, $D_j$, was calculated using Eqn. 5. Where $p$ is the number of parameters, and $w_i$ is the assigned weight of parameter $i$ (Table 2).

Eqn. 4 $$d_{ij} = \frac{\mu_{ij} - LB_i}{UB_i - LB_i}$$

Eqn. 5 $$d_{ij} = \frac{UB_i - \mu_{ij}}{UB_i - LB_i}$$

Eqn. 2 $$UB_i = max(\mu_{i:}) + 1\% \times \left(max(\mu_{i:}) - min(\mu_{i:})\right)$$

Eqn. 3 $$LB_i = min(\mu_{i:}) - 1\% \times \left(max(\mu_{i:}) - min(\mu_{i:})\right)$$

Eqn. 1 $$D_j = \left(\prod_{1}^{p} d_{ij}^{w_i}\right)^{\frac{1}{\sum_1^p w_i}}$$

### Pooled serum pseudovirus neutralization assay

To test for neutralizing antibodies, non-replicating lentiviral particles expressing the SARS-CoV-2 spike variant proteins on their envelope membrane and encoding luciferase as a reporter were prepared. Infection was quantified using in vitro-grown human 293T-hACE-2 cells based on luciferase expression. The pseudovirus production system included the luciferase-encoding reporter plasmid, pNL4-3.LucR-E-, a Gag/Pol-encoding packaging construct (pΔ8.9), and the codon-optimized SARS-CoV-2 spike VOC-expressing plasmids (pcDNA3.1-CoV-2 S gene), based on clone p278-1 (Pollet et al., 2021). Pseudovirus-containing supernatants were recovered 48 h after transfection, passed through a 0.45-µm filter, and saved at −80 °C until used for neutralization studies.

The expression plasmids for SARS-CoV-2 spike variants were generated by site-directed mutagenesis or replacement of segments of the codon-optimized Wuhan SARS-CoV-2 spike expression clone, p278-1, with variant sequences as previously described (Pollet et al., 2022). The sequences of all the variant spike genes were confirmed via commercial DNA sequencing.

The pseudovirus assay was performed as described earlier (Pollet et al., 2021). Briefly, 10 µL of pseudovirus were incubated with serial dilutions of the serum samples for 1 h at 37 °C. Next, 100 µL of sera-pseudovirus were added to 293T-hACE-2 cells in 96-well poly-D-lysine-coated culture plates. Following 48 h of incubation in a 5% $CO_2$ environment at 37 °C, the cells were lysed with 100 µL of Promega Glo Lysis buffer for 15 min at room temperature. Finally, 50 µL of the lysate was added to 50 µL luciferase substrate (Promega Luciferase Assay System). The amount of luciferase was quantified with luminescence (relative luminescence units (RLU)), using the Luminometer (Biosynergy H4, BioTek). Sera from vaccinated mice were compared by their 50% inhibitory dilution (IC50), defined as the serum dilution at which the virus infection was reduced by 50% compared to the negative control (virus + cells). IC50 values were calculated as described by Nie et al. (Nie et al., 2020).

### Sodium dodecyl sulfate–polyacrylamide gel electrophoresis (SDS-PAGE)

RBD vaccines were formulated as described in the main text and allowed to incubate at RT for 1 h. 2 µg of protein were loaded per well onto a 4-20% Tris-glycine gel (Invitrogen #XP04200BOX), using a Mark12 reference standard (Invitrogen #LC5677). Samples were run at 180 V for 65 min and stained using the SPYRO Ruby gel stain kit overnight. Densitometry measurements were performed using a BioRad ChemiDoc MP imaging system.

## 2 SUPPLEMENTARY REFERENCES

Nie, J., Li, Q., Wu, J., Zhao, C., Hao, H., Liu, H., et al. (2020). Quantification of SARS-CoV-2 neutralizing antibody by a pseudotyped virus-based assay. *Nat Protoc* 15, 3699–3715. doi: 10.1038/s41596-020-0394-5.

Pollet, J., Chen, W.-H., Versteeg, L., Keegan, B., Zhan, B., Wei, J., et al. (2021). SARS-CoV-2 RBD219-N1C1: A yeast-expressed SARS-CoV-2 recombinant receptor-binding domain candidate vaccine stimulates virus neutralizing antibodies and T-cell immunity in mice. *Human Vaccines & Immunotherapeutics* 17, 2356–2366. doi: 10.1080/21645515.2021.1901545.

Pollet, J., Strych, U., Chen, W.-H., Versteeg, L., Keegan, B., Zhan, B., et al. (2022). Receptor-binding domain recombinant protein on alum-CpG induces broad protection against SARS-CoV-2 variants of concern. *Vaccine* 40, 3655–3663. doi: 10.1016/j.vaccine.2022.05.007.

# 3 SUPPLEMENTARY FIGURES

| Procedure/Study Day | -7 | -1 | 0 | 20 | 21 | 42 |
|---|---|---|---|---|---|---|
| Arrival | X | | | | | |
| Marking of ID and allocation to groups | X | | | | | |
| Immunization | | | X | | X | |
| Blood sample | | X | | X | | X |
| BAL, bone marrow, and spleen harvest | | | | | | X |
| Termination | | | | | | X |

**Supplementary Figure S1: Study outline for all murine immunogenicity studies.**

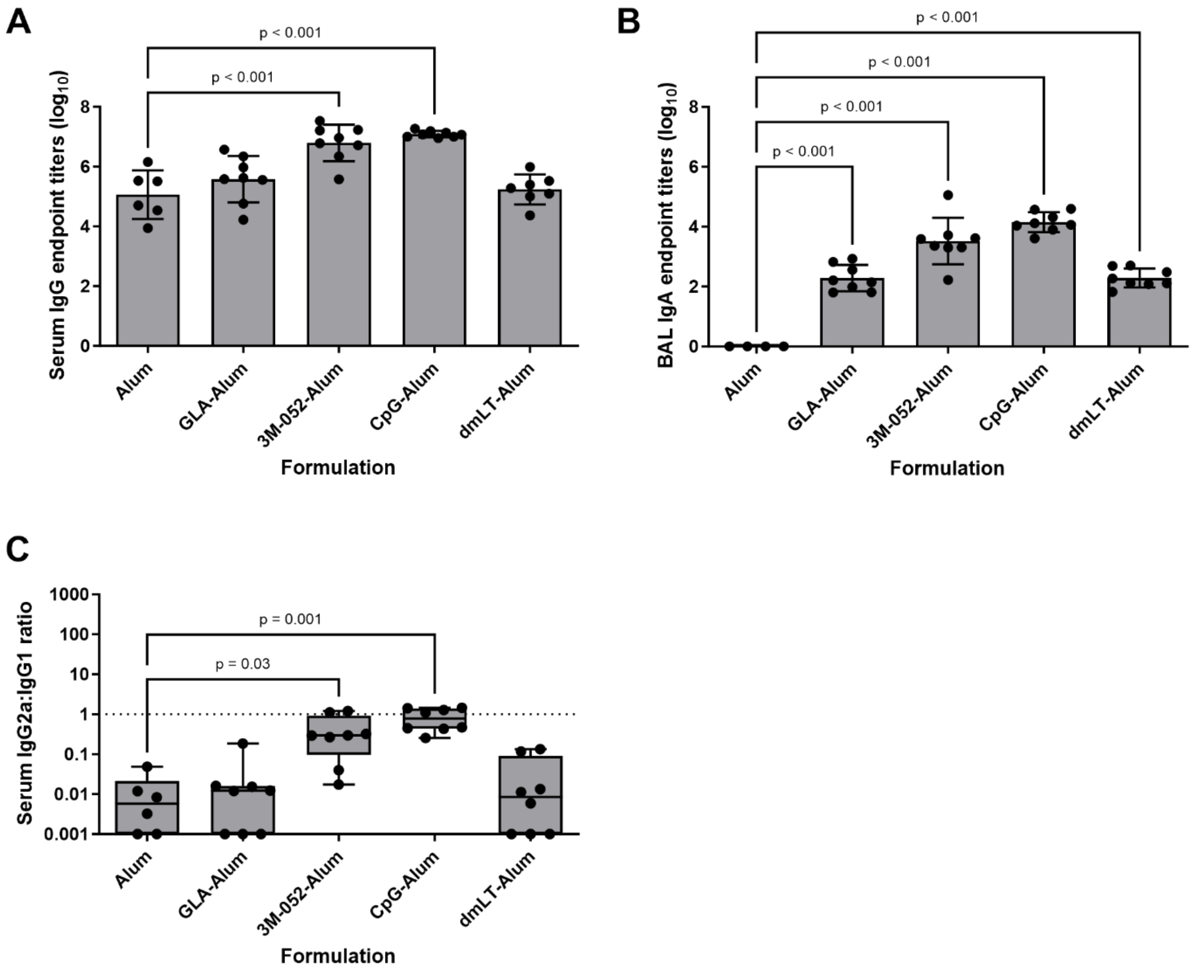


**Supplemental Figure S2: Data from Figure 1, only showing comparisons between groups that received vaccines formulated with Alum.** Data collected from animals ($n$ = 8 per group, 4M:4F) vaccinated twice intramuscularly with RBD in combination with the indicated adjuvant formulation (Day 0 and Day 21). All samples and data in this figure were collected and processed simultaneously. (A) Serum titer of total anti-RBD IgG, (B) bronchoalveolar lavage (BAL) titer of anti-RBD IgA, and (C) serum ratio of exponentiated anti-RBD IgG2a/IgG1 titers 42 days post-prime. Note: Only comparisons to Alum without the addition of agonist are shown. (A, B) Statistical significance was determined via one-way ANOVA followed by a Holm-Sidak's correction for multiple comparisons fixing the family-wide error rate to 0.05. Bars represent the mean ± SD of log-normalized data. (C) Statistical significance was determined via a non-parametric Kruskal-Wallis test followed by a Dunn's correction for multiple comparisons fixing the family-wide error rate to 0.05. Data are represented as box-whisker plots with bars representing median values, boxes representing 1st-3rd quartiles, and whiskers representing the maximum and minimum values.

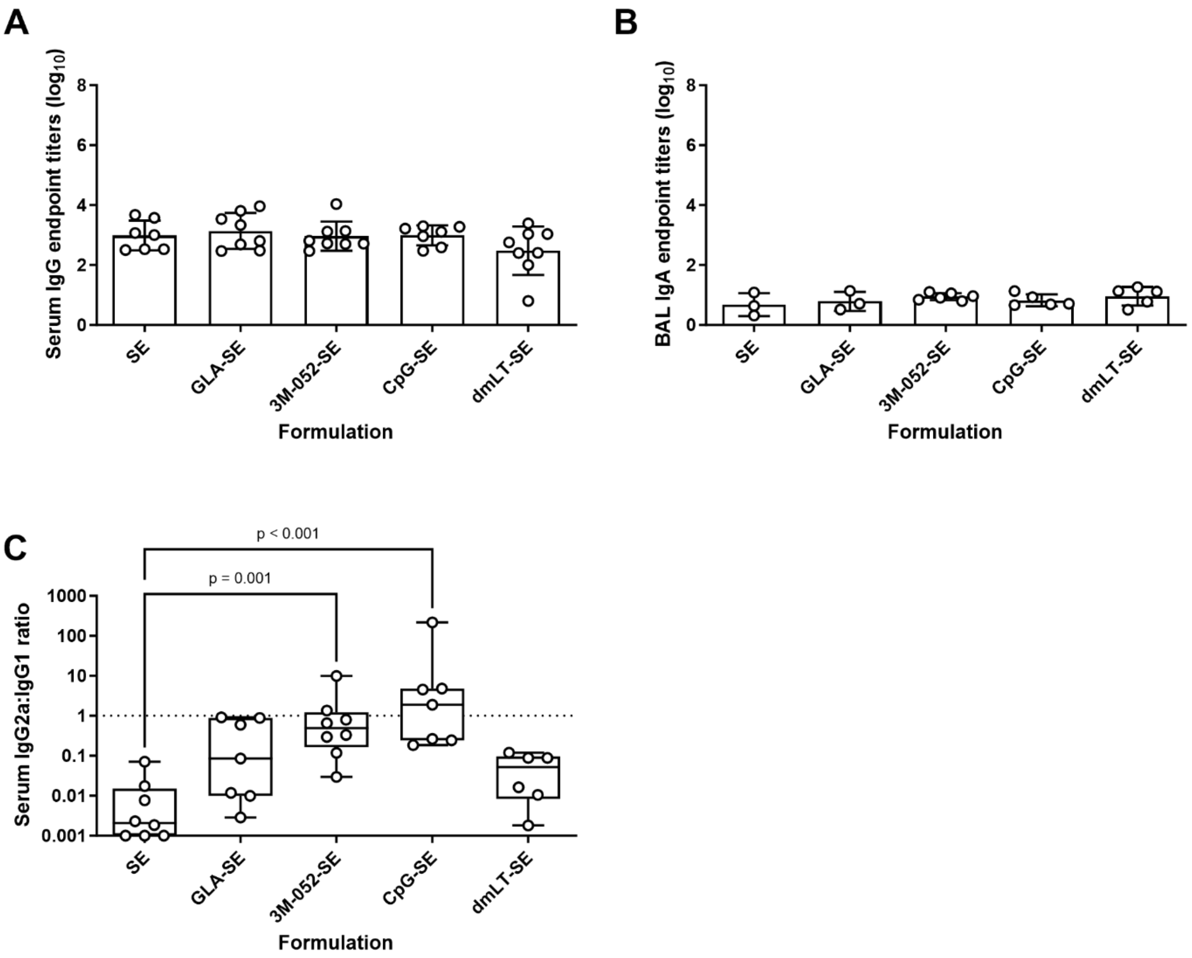


**Supplemental Figure S3: Data from Figure 1, only showing comparisons between groups that received vaccines formulated with SE.** Data collected from animals ($n$ = 8 per group, 4M:4F) vaccinated twice intramuscularly with RBD in combination with the indicated adjuvant formulation (Day 0 and Day 21). All samples and data in this figure were collected and processed simultaneously. (A) serum titer of total anti-RBD IgG, (B) BAL titer of anti-RBD IgA, and (C) serum ratio of exponentiated anti-RBD IgG2a/IgG1 titers 42 days post-prime. Note: Only comparisons to SE without the addition of agonists are shown. (A, B) Statistical significance was determined via one-way ANOVA followed by a Holm-Sidak's correction for multiple comparisons fixing the family-wide error rate to 0.05. Bars represent the mean ± SD of log-normalized data. (C) Statistical significance was determined via a non-parametric Kruskal-Wallis test followed by a Dunn's correction for multiple comparisons fixing the family-wide error rate to 0.05. Data are represented as box-whisker plots with bars representing median values, boxes representing 1st-3rd quartiles, and whiskers representing the maximum and minimum values.

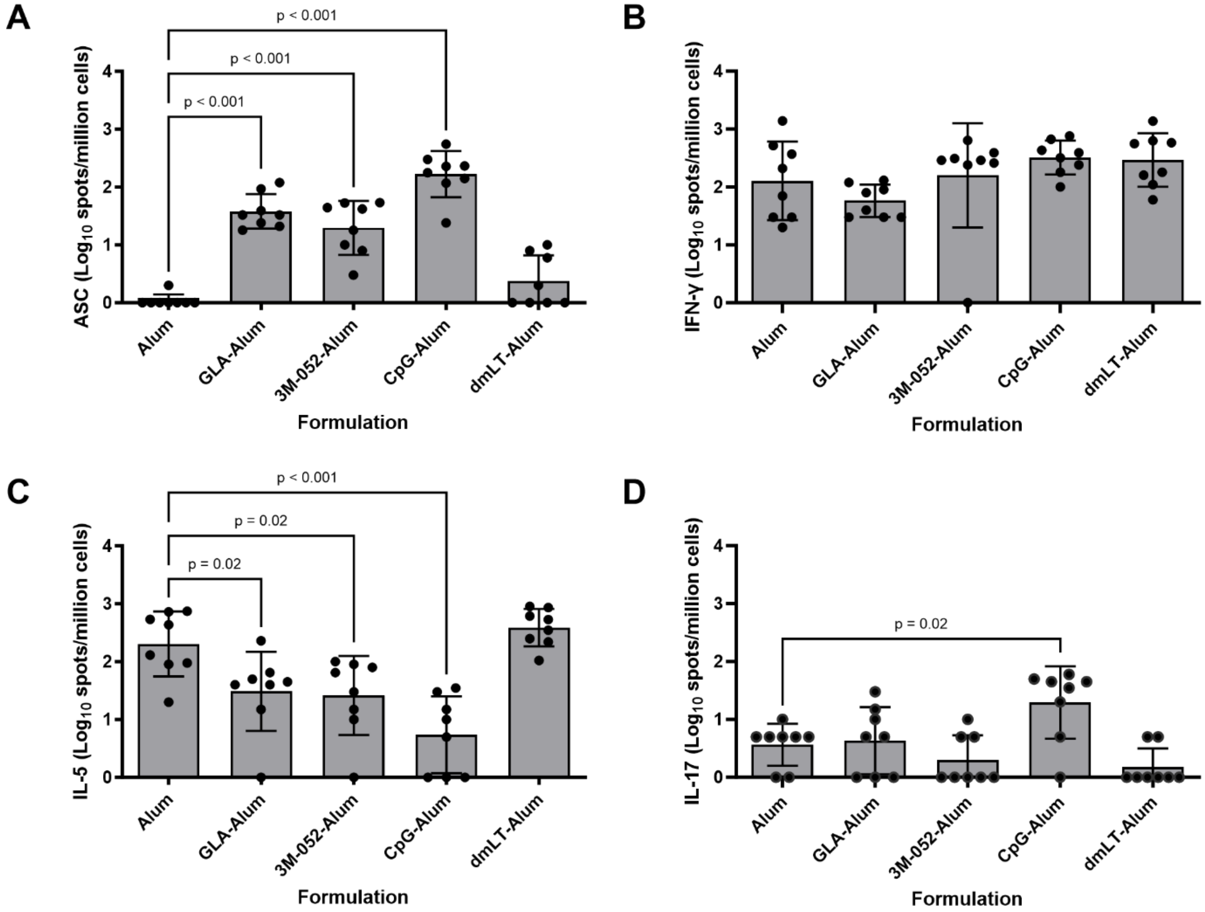


**Supplementary Figure S4: Data from Figure 2, only showing comparisons between groups that received vaccines formulated with Alum.** Data collected from animals (*n* = 8 per group, 4M:4F) vaccinated twice intramuscularly with RBD in combination with the indicated adjuvant formulation (Day 0 and Day 21). All samples and data in this figure were collected and processed simultaneously. (A) Bone marrow-derived anti-spike IgG antibody-secreting cells (ASC) ELISpot. ELISpot measurement of splenocytes secreting (B) IFN-γ, (C) IL-5, or (D) IL-17 upon stimulation with a SARS-CoV-2 peptide pool 42 days post-prime. Statistical significance was determined via a two-way ANOVA with a full effects model test followed by Holm-Sidak's correction for multiple comparisons, fixing the family-wide error rate to 0.05. Only comparisons to Alum without the addition of an agonist are shown. Bars represent the mean ± SD of log-normalized data.

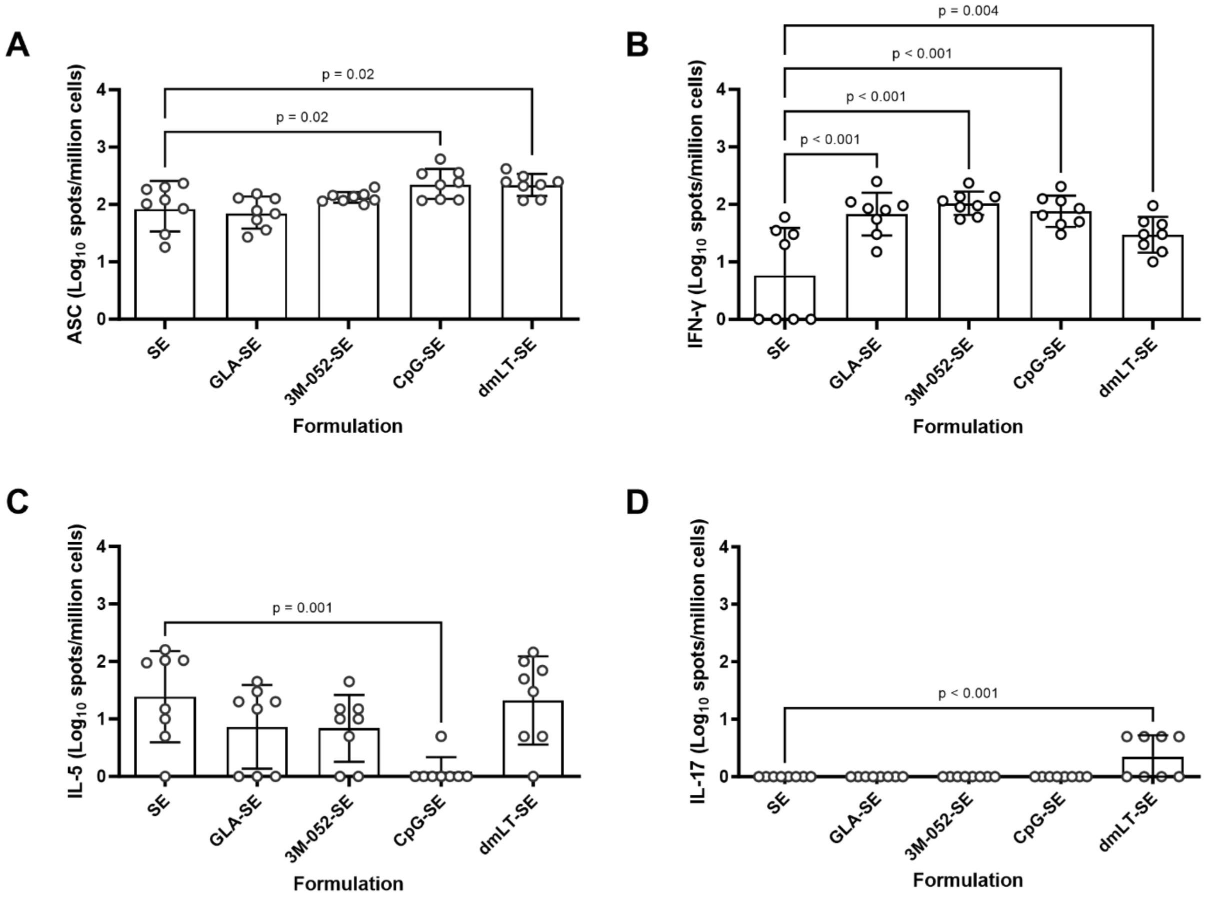


**Supplementary Figure S5: Data from Figure 2, only showing comparisons between groups that received vaccines formulated with SE.** Data collected from animals (*n* = 8 per group, 4M:4F) vaccinated twice intramuscularly with RBD in combination with the indicated adjuvant formulation (Day 0 and Day 21). All samples and data in this figure were collected and processed simultaneously. (A) Bone marrow-derived anti-spike IgG antibody-secreting cells (ASC) ELISpot. ELISpot measurement of splenocytes secreting (B) IFN-γ, (C) IL-5, or (D) IL-17 upon stimulation with a SARS-CoV-2 peptide pool 42 days post-prime. Statistical significance was determined via a two-way ANOVA with a full effects model test followed by Holm-Sidak's correction for multiple comparisons, fixing the family-wide error rate to 0.05. Only comparisons to SE without the addition of an agonist are shown. Bars represent the mean ± SD of log-normalized data.

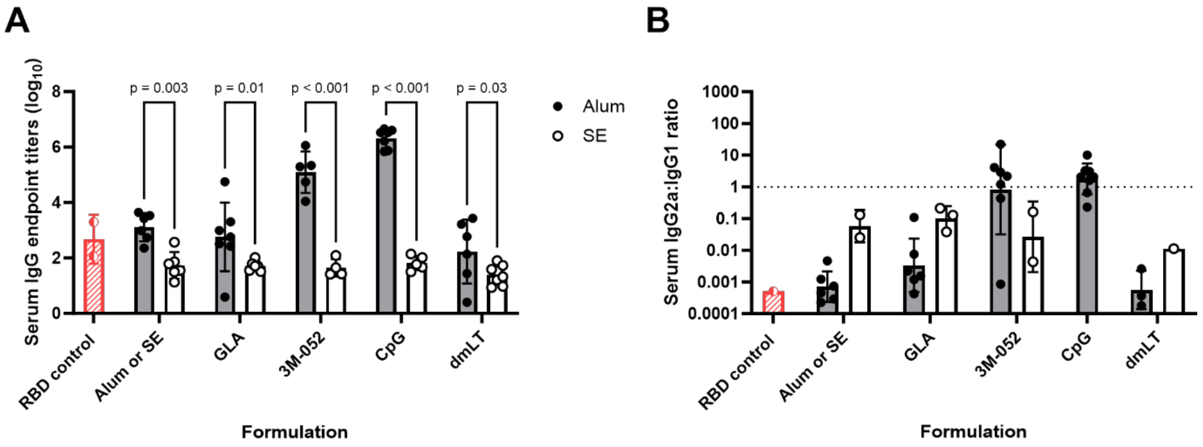


**Supplementary Figure S6: Humoral responses 21 days post-prime.** Mice (*n* = 8 per group, 4M:4F) were vaccinated once with RBD in combination with the indicated receptor agonist formulated with either Alum or SE. Serum samples were collected via retro-orbital bleed 21 days post-prime. RBD control data (red striped) collected from animals that were vaccinated with RBD without the addition of an adjuvant. Study was divided in half and vaccinations/harvests were staggered 1 week apart to reduce operator burden. Assays presented here were performed for all animals simultaneously using frozen serum samples. Animals in the Alum-containing groups and the RBD control group were vaccinated and harvested on different days than those in the SE-containing groups. (A) Serum titer of total anti-RBD IgG and (B) ratio of exponentiated serum anti-RBD IgG2a/IgG1 titers. (A) Statistical significance was determined via repeated *t*-tests followed by a Holm-Sidak's correction for multiple comparisons fixing the family-wide error rate to 0.05. Horizontal lines represent the mean ± SD of log-normalized data. (B) Statistical significance was determined via multiple non-parametric Mann-Whitney tests followed by a Holm-Sidak's correction for multiple comparisons fixing the family-wide error rate to 0.05. Horizontal lines represent the geometric mean ± geometric SD.

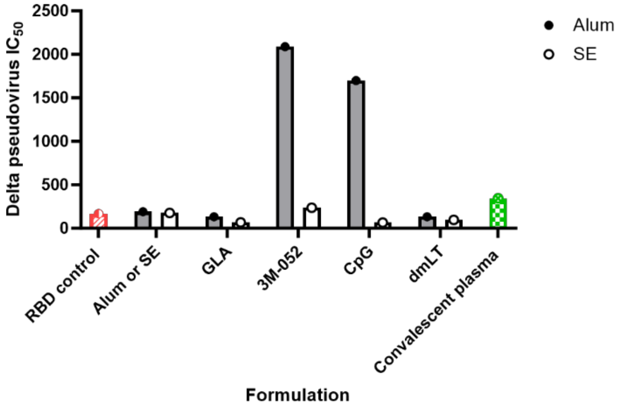


**Supplementary Figure S7: Pseudovirus neutralization assay of pooled mouse serum.** Data collected from animals (*n* = 8 per group, 4M:4F) vaccinated twice with RBD in combination with the indicated receptor agonist formulated with either Alum or SE (Day 0 and Day 21). RBD control data (red striped) collected from animals that were vaccinated with RBD without the addition of an adjuvant. Convalescent plasma (green checkered) obtained from the Frederick National Laboratory (Rockville, MD, COVID-NS01097). Each data point is an *n* = 1 replicate from a serum sample pooled from 6 to 8 animals per group 42 days post-prime. Neutralization measured against a Delta variant pseudovirus (B.1.617.2). Study was divided in half and vaccinations/harvests were staggered 1 week apart to reduce operator burden. The assay presented here was performed for all animals simultaneously using frozen serum samples. Animals in the Alum-containing groups and the RBD control group were vaccinated and harvested on different days than those in the SE-containing groups.

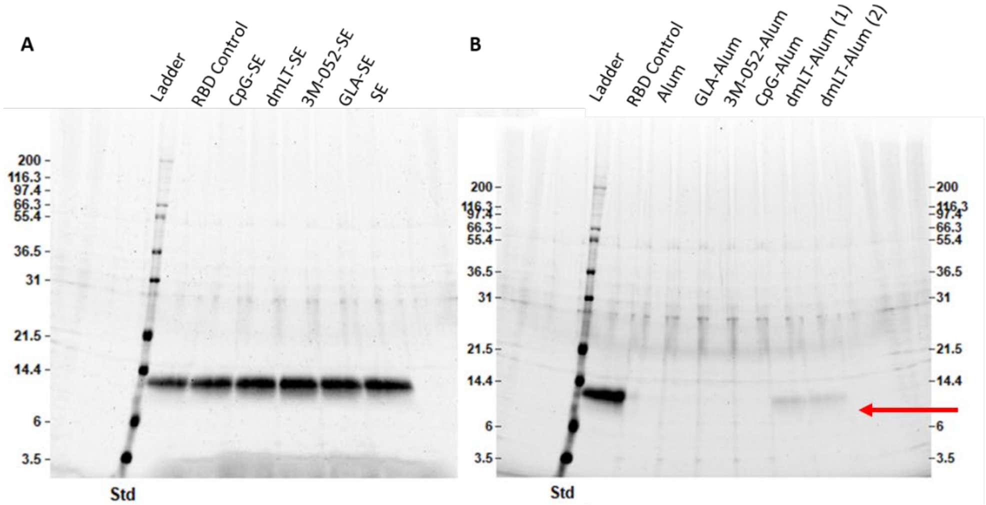


C

| Formulation | Relative Quantity |
|---|---|
| CpG-SE | 1.35 |
| dmLT-SE | 1.54 |
| 3M-052-SE | 1.64 |
| GLA-SE | 1.58 |
| SE | 1.60 |
| Alum | 0 |
| GLA-Alum | 0 |
| 3M-052-Alum | 0 |
| CpG-Alum | 0 |
| dmLT-Alum (1) | 0.06 |
| dmLT-Alum (2) | 0.04 |

**Supplementary Figure S8: SDS-PAGE analysis of the vaccine formulations.** (A) SE-formulated vaccines, (B) Alum-formulated vaccines (red arrow highlights unbound dmLT), and (C) relative densitometry measurements (to RBD controls in each gel). RBD control represents RBD without the addition of an adjuvant. dmLT-Alum was run twice to confirm residual unbound protein content (see red arrow).

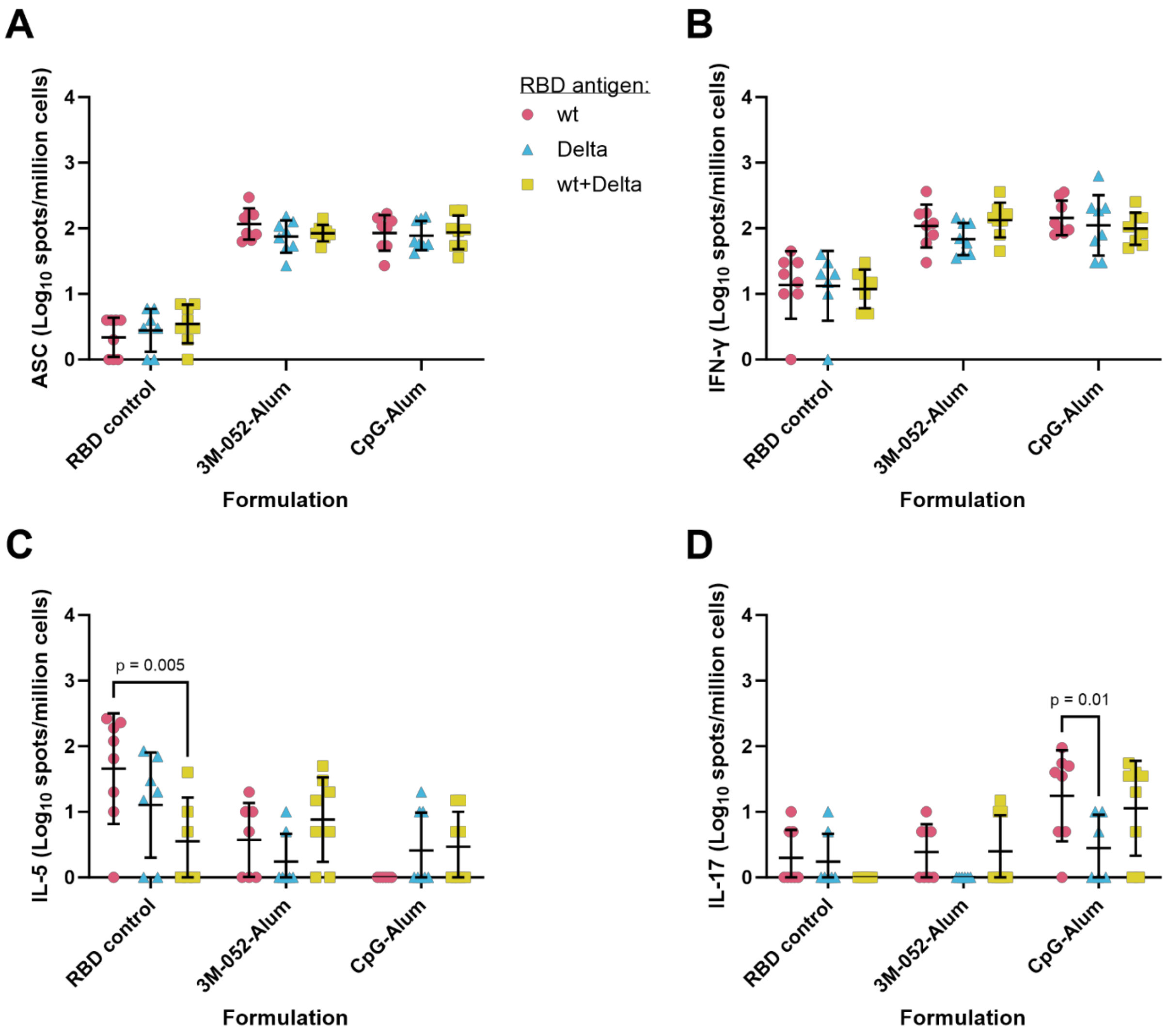


**Supplementary Figure S9: ELISpot responses from mice that received an adjuvanted multivalent RBD vaccine.** Data collected from mice (*n* = 8 per group, 4M:4F) vaccinated twice intramuscularly (Day 0 and Day 21). Spleen and bone marrow tissue harvested 42 days post-prime. Mice were vaccinated with the indicated RBD: wt RBD, Delta RBD, or wt+Delta RBD formulated with the indicated adjuvant. RBD control data were collected from animals that were vaccinated with the indicated RBD strain(s) without the addition of an adjuvant. Study groups were divided evenly in half and vaccinations/harvests were staggered 1 week apart to reduce operator burden. Assays presented here were performed at the time of tissue harvest. (A) Bone marrow-derived anti-full-length-wt-spike IgG antibody-secreting cells (ASC) ELISpot. ELISpot measurement of splenocytes secreting (B) IFN-γ, (C) IL-5, or (D) IL-17 upon stimulation with a SARS-CoV-2 peptide pool. Statistical significance was determined via two-way ANOVA followed by Holm-Sidak's correction for multiple comparisons, fixing the family-wide error rate to 0.05. Horizontal lines represent the mean ± SD of log-normalized data.

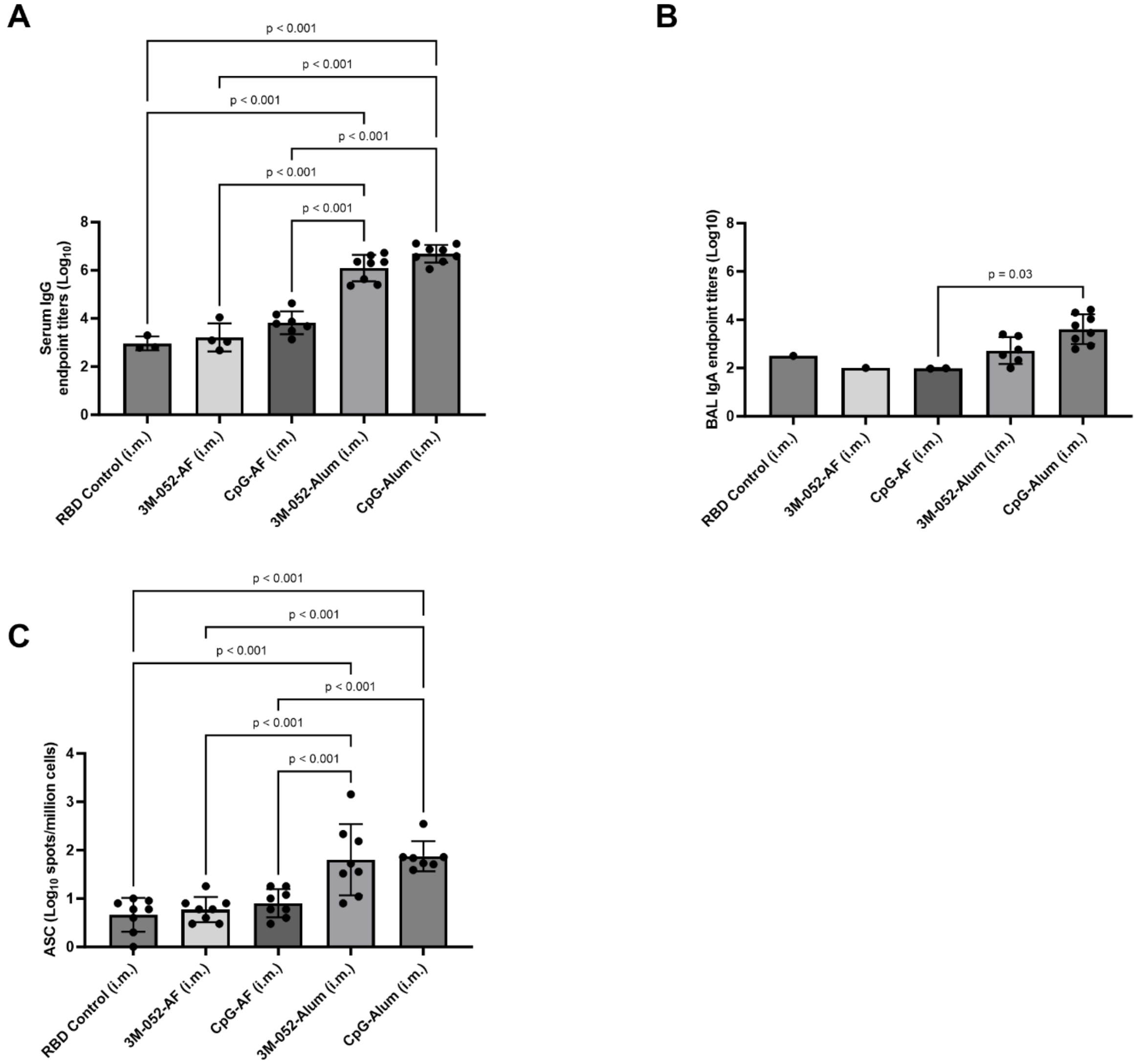


**Supplementary Figure S10: Alum improves the immunogenicity and response rate of the RBD antigen formulated with 3M-052 or CpG.** Data collected from mice (*n* = 8 per group, 4M:4F) vaccinated twice intramuscularly (i.m.) on Day 0 and Day 21. Serum, BAL, and bone marrow samples were harvested 42 days post-prime. Mice were vaccinated with the wt RBD formulated with the indicated adjuvant. RBD control data were collected from animals that were vaccinated with the wt RBD strain without the addition of an adjuvant. Study groups were divided evenly in half and vaccinations/harvests were staggered 1 week apart to reduce operator burden. Assays in panels (A) and (B) were performed for all animals simultaneously using frozen serum and BAL samples; assay in panel (C) was performed at the time of tissue harvest. (A) Serum titer of total anti-RBD IgG, (B) BAL titer of anti-RBD IgA, (C) Bone marrow-derived anti-full-length-wt-spike IgG antibody-secreting cells (ASC) ELISpot. Statistical significance was determined via one-way ANOVA followed by Holm-Sidak’s correction for multiple comparisons, fixing the family-wide error rate to 0.05. Horizontal lines represent the mean ± SD of log-normalized data. AF = aqueous formulation (not formulated with Alum or SE).
